# The role of B lymphocyte subsets in nephrotic syndrome: functions, mechanisms, clinical significance and future perspectives

**DOI:** 10.3389/fimmu.2025.1598197

**Published:** 2025-08-07

**Authors:** Yuheng Liao, Haofei Hu, Qijun Wan, Haiying Song

**Affiliations:** ^1^ Department of Nephrology, Shenzhen Second People’s Hospital, Shenzhen, Guangdong, China; ^2^ School of Medicine Shenzhen University, Shenzhen, China; ^3^ Department of Nephrology, The First Affiliated Hospital of Shenzhen University, Shenzhen, Guangdong, China

**Keywords:** B lymphocytes, nephrotic syndrome, immune regulation, biomarkers, precision medicine

## Abstract

B lymphocytes play a critical role in the pathogenesis of nephrotic syndrome (NS). This comprehensive review explores the phenotypic characteristics, pathogenic mechanisms, and clinical translational value of B cell subsets in different types of nephrotic syndrome. Studies demonstrate that B cells participate in disease development through multiple mechanisms, including autoantibody production, T cell function regulation, and cytokine secretion. In minimal change disease, B cell-mediated immune dysregulation is primarily characterized by decreased CD19+ cells and increased plasmablasts. Membranous nephropathy patients exhibit increased naïve B cells and decreased memory B cells, while focal segmental glomerulosclerosis is characterized by elevated class-switched memory B cells. These B cell subset alterations can serve as biomarkers for disease activity assessment and prognosis prediction. B cell-targeted therapies, such as anti-CD20 monoclonal antibodies, have demonstrated significant therapeutic efficacy in nephrotic syndrome, further confirming the pivotal role of B cells in its pathogenesis. Different pathological types of NS show significant differences in B cell subset changes, pathogenic mechanisms, and therapeutic responses. Primary and secondary nephrotic syndrome exhibit important distinctions in B cell activation mechanisms, subset imbalance patterns, degree of renal tissue infiltration, and autoantibody profiles. Age factors significantly influence B cell development, function, and therapeutic response, with notable differences between pediatric and adult patients in B cell subset distribution, treatment efficacy, and pharmacokinetics. With the application of emerging technologies such as single-cell sequencing, in-depth analysis of B cell subset characteristics and their interactions with other immune cells will provide new insights for developing more precise diagnostic and therapeutic strategies. However, current methodological heterogeneity challenges in research, including patient population differences, inconsistent B cell subset definitions, technical platform variations, and non-uniform clinical assessment criteria, limit the comparability of research results and clinical applications. Future efforts need to establish standardized B cell monitoring protocols and precision diagnostic systems, develop next-generation B cell-targeted therapeutic strategies, and deeply explore age-specific mechanisms and systems biology research to achieve precision medicine in nephrotic syndrome.

## 1 Introduction

Nephrotic syndrome (NS) is an autoimmune disorder characterized by podocyte foot process effacement due to an altered glomerular filtration barrier, leading to severe proteinuria, hypoalbuminemia, and edema. In recent years, the potential role of B cells in NS has gained increasing attention, particularly following the remarkable efficacy of B cell depletion therapy (such as the anti-CD20 monoclonal antibody rituximab (RTX)) in inducing and maintaining long-term remission in NS patients (1). Studies have shown that pathogenic B cells not only alter immune homeostasis through antibody production but may also directly affect podocyte structure and function through activation of surface molecules or secretion of specific cytokines, or by modulating T cell homeostasis, ultimately resulting in renal injury (2).

B lymphocytes comprise heterogeneous subsets with distinct phenotypes and functions. Abnormalities in their differentiation and development may lead to B cell dysregulation, triggering various autoimmune diseases, including nephrotic syndrome (3, 4). Research has revealed that patients with membranous nephropathy (MN) exhibit significantly increased numbers of plasma cells and regulatory B cells compared to healthy individuals (5). Furthermore, Ling et al. discovered significantly elevated transitional B cell counts in steroid-sensitive nephrotic syndrome (SSNS) patients and proposed this as a potential biomarker for early SSNS screening (6). Additional studies have suggested associations between NS and B cell-derived malignancies such as Hodgkin’s and non-Hodgkin’s lymphomas (7–9) and Epstein-Barr virus infection, which primarily targets B cells (10). These findings provide new perspectives on the pathogenesis of NS.

Recent advances have significantly improved our understanding of the clinicopathologic characteristics of both primary and secondary NS subtypes, each presenting distinct pathophysiological profiles that may influence B cell subset involvement. Contemporary studies have revealed new insights into the pathogenesis and clinical management of these conditions, with comprehensive analyses of adult minimal change disease (MCD) and focal segmental glomerulosclerosis (FSGS) providing crucial clinical insights (11, 12). MCD remains an important cause of NS across age groups, with recent studies highlighting novel therapeutic approaches including RTX therapy even in patients without detectable B cells, and successful treatment of refractory cases (13, 14). FSGS represents a spectrum of podocyte disorders with emerging evidence for immune-mediated mechanisms, including studies of patients presenting with kidney function loss (15). MN is now recognized as an antibody-mediated autoimmune disease with recent breakthroughs including environmental factor research (16). Understanding these distinct pathophysiological mechanisms is crucial for elucidating the specific contributions of B lymphocyte subsets to disease pathogenesis and developing targeted therapeutic approaches.

Although the role of B cells in the immunopathogenesis of NS has received increasing attention, research on the alterations, mechanisms, and clinical significance of specific B cell subsets remains limited. Therefore, this review integrates existing evidence to outline the roles of B cell subsets in different pathological types of NS and discusses relevant B cell-targeted therapeutic strategies.

## 2 Materials and methods

This comprehensive narrative review was conducted to synthesize current knowledge on B lymphocyte subsets in nephrotic syndrome pathogenesis, mechanisms, and therapeutic applications.

### 2.1 Literature search strategy

A systematic literature search was performed using “PubMed/MEDLINE”, “Web of Science Core Collection”, “Cochrane Library” from 1974 to 2025. The extended timeframe ensured inclusion of seminal foundational studies alongside the most recent advances in B cell immunology.

Search terms included combinations of: “nephrotic syndrome”; “B cells”; “B lymphocytes”; “memory B cells”; “plasma cells”; “plasmablasts”; “regulatory B cells”; “rituximab”; “CD20”; “minimal change disease”; “membranous nephropathy”; “focal segmental glomerulosclerosis”; “membranoproliferative glomerulonephritis”; “lupus nephritis”; “diabetic nephropathy” and “ hepatitis B-related nephropathy.”

### 2.2 Study selection and inclusion criteria

Studies were selected based on relevance to B cell biology in nephrotic syndrome, methodological quality, and contribution to mechanistic understanding or therapeutic approaches. Inclusion criteria encompassed: Original research investigating B cell subpopulations in NS; Clinical trials evaluating B cell-targeted therapies; Studies utilizing standardized immunophenotyping methods; Research with appropriate control groups and adequate sample sizes; Publications in peer-reviewed journals with clear outcome measures

### 2.3 Data extraction and synthesis

This narrative review synthesized findings from 243 selected references, including prospective and retrospective studies, randomized controlled trials, meta-analyses, and single-cell sequencing investigations. Data extraction focused on study design, patient demographics, B cell analysis methods, key findings, and clinical implications. Given the heterogeneity of study populations (pediatric vs. adult) and methodological approaches, a qualitative synthesis framework was employed to integrate findings and identify key themes in B cell-mediated NS pathogenesis.

## 3 Clinicopathological and immunological features of nephrotic syndrome

### 3.1 Clinicopathological features of major nephrotic syndrome

#### 3.1.1 Minimal change disease

Recent comprehensive analysis of adult MCD has provided crucial insights into its distinct clinicopathologic characteristics (11):

##### 3.1.1.1 Clinical features

1. Adult MCD represents 10-20% of adult nephrotic syndrome cases, with distinct characteristics compared to pediatric presentations; Sudden onset of massive proteinuria with relatively preserved glomerular filtration rate in most patients;
2. Higher risk of complications and different treatment response patterns compared to pediatric cases (11);
3. Novel Insights from Recent Research: Adult patients demonstrate increased susceptibility to thromboembolic complications and acute kidney injury compared to children, with steroid response rates of 80-85% versus >95% in pediatric populations;
4. Association with medications, infections, and rarely malignancies in adult presentations;
5. Some cases may present as refractory disease requiring alternative therapeutic approaches;
6. Advanced Understanding (14): Recent studies reveal that adult MCD patients with comorbid conditions like β-thalassemia and autoimmune hemolytic anemia can achieve successful outcomes with rituximab therapy, even in previously treatment-resistant cases;
7. Enhanced Mechanistic Understanding (13): Even in patients with undetectable B cells in peripheral circulation, rituximab therapy can induce complete remission through depletion of CD20+ T lymphocytes, revealing novel immune mechanisms beyond traditional B cell paradigms.

##### 3.1.1.2 Pathological features

1. Normal glomerular morphology under light microscopy with minimal mesangial changes;
2. Absence of immune complex deposits on immunofluorescence;
3. Diffuse foot process effacement (>80%) on electron microscopy as the hallmark feature;
4. Recent ultrastructural studies indicate potential podocyte cytoskeletal abnormalities involving nephrin and podocin expression.

#### 3.1.2 Focal segmental glomerulosclerosis

Comprehensive analysis of FSGS has revealed crucial insights into this spectrum of podocyte disorders with emerging evidence for immune-mediated mechanisms (12, 15):

##### 3.1.2.1 Clinical pathology and histologic variants

FSGS represents a leading cause of primary glomerular disease with heterogeneous presentations. Detailed pathological analysis has identified distinct variants with significantly different clinical outcomes (12):

1. Tip variant: Better treatment response and improved long-term prognosis, with complete remission rates of 65% compared to 35% in other variants;
2. Collapsing variant: Most aggressive course with rapid progression to end-stage renal disease;
3. Cellular variant: Intermediate prognosis with moderate steroid responsiveness (45% remission rate);
4. Perihilar variant: Often associated with adaptive mechanisms secondary to reduced nephron mass;
5. Not otherwise specified (NOS): Most common variant (60% of cases) with variable outcomes depending on clinical presentation.

##### 3.1.2.2 Clinical course in patients with kidney function loss

Enhanced analysis based on recent comprehensive studies of patients presenting with elevated creatinine (15):

1. Patients presenting with kidney function loss (serum creatinine ≥1.5 mg/dL) show distinct clinical patterns with significantly worse long-term outcomes;
2. Kidney function loss on presentation represents the most important prognostic factor, with 5-year renal survival rates of 50% versus 85% in patients with preserved initial function;
3. These patients demonstrate higher rates of treatment resistance (70% vs 40%) and faster progression to chronic kidney disease;
4. Progressive decline varies based on histologic variant and initial presentation, with collapsing variant showing the most rapid deterioration even among those with preserved initial function;
5. Treatment response correlates strongly with both variant type and degree of initial kidney function impairment, necessitating variant-specific therapeutic approaches;
6. New mechanistic insights suggest that early kidney function loss reflects underlying podocyte depletion and immune-mediated injury that may be partially reversible with aggressive B cell-targeted therapies.

#### 3.1.3 Membranous nephropathy

Membranous nephropathy is now recognized as an antibody-mediated autoimmune disease with significant recent breakthroughs (16):

##### 3.1.3.1 Clinical features

1. Leading cause of adult nephrotic syndrome in Caucasian populations;
2. Peak incidence in middle-aged adults with gradual onset of proteinuria and edema;
3. Rule of thirds: 30% spontaneous remission, 30% stable proteinuria, 30% progression;
4. Environmental factors may significantly influence disease development and progression (16): Recent groundbreaking research demonstrates that PM2.5 exposure induces oxidative stress leading to upregulated PLA2R expression in lung tissue, with subsequent extracellular vesicle-mediated transport to kidneys, establishing the first direct environmental-immunological link in MN pathogenesis.

##### 3.1.3.2 Pathological features

1. Characteristic subepithelial immune complex deposits creating ‘spike and dome’ appearance on electron microscopy;
2. Diffuse granular IgG and C3 deposition along glomerular basement membrane on immunofluorescence;
3. Stage-dependent prognosis based on the extent of tubular atrophy and interstitial fibrosis;
4. GBM thickening progressively increasing with disease duration;
5. Novel Immunological Variants (15): Rare cases of IgG4-related disease can present with IgG1-dominant rather than typical IgG4-dominant membranous nephropathy, suggesting alternative immune complex formation mechanisms and distinct B cell activation pathways.

##### 3.1.3.3 Advanced environmental and immunological mechanisms

Comprehensive analysis reveals complex interactions between environmental factors and immune dysregulation (16):

1. PM2.5-induced oxidative stress creates a systemic inflammatory environment that promotes molecular mimicry and cross-reactive antibody production;
2. Extracellular vesicles serve as vehicles for antigen transport from lung to kidney, establishing a novel environmental-renal disease axis;
3. This environmental trigger may explain geographic variations in MN incidence and the increasing prevalence in industrialized regions.

#### 3.1.4 Mesangioproliferative glomerulonephritis

Mesangioproliferative glomerulonephritis (MsPGN) represents a heterogeneous group of glomerular diseases characterized by mesangial proliferation (17):

##### 3.1.4.1 Clinical features

1. More common in children and young adults with variable presentations;
2. Clinical spectrum ranges from asymptomatic proteinuria to full nephrotic syndrome;
3. Frequently accompanied by microscopic hematuria and mild hypertension;
4. Variable progression rate with some cases showing spontaneous improvement.

##### 3.1.4.2 Pathological features

1. Diffuse mesangial hypercellularity with increased mesangial matrix deposition;
2. Mesangial immune complex deposits, predominantly IgM and C3 on immunofluorescence;
3. Electron microscopy reveals mesangial electron-dense deposits;
4. May overlap with other proliferative patterns.

### 3.2 Enhanced B cell mechanistic integration

Integration with B Cell Biology:
The enhanced understanding of these clinicopathologic characteristics provides crucial context for B lymphocyte subset involvement:

#### 3.2.1 Minimal change disease

1. The paradoxical effectiveness of rituximab even in B cell-depleted patients (13) suggests that CD20+ T cells may represent a previously unrecognized pathogenic population;
2. Successful treatment of complex cases with comorbid autoimmune conditions (14) indicates that B cell depletion can break pathogenic immune cascades involving multiple cell types;
3. These findings necessitate revision of purely B cell-centric disease models to include broader immune network dysfunction;

#### 3.2.2 Focal segmental glomerulosclerosis

1. The variant-specific outcomes (12) may reflect distinct patterns of B cell activation and antibody production, with tip variant showing better response potentially due to different immune activation profiles;
2. Early kidney function loss (15) could represent a threshold of immune-mediated damage where B cell-targeted intervention becomes less effective, requiring earlier and more aggressive immunosuppression;
3. The heterogeneous treatment responses suggest that precision medicine approaches targeting specific B cell subsets based on variant type may improve outcomes.

#### 3.2.3 Membranous nephropathy

1. Environmental triggers like PM2.5 (16) provide the first direct link between environmental exposure and B cell activation in autoimmune kidney disease;
2. The extracellular vesicle-mediated antigen transport mechanism represents a novel pathway for environmental antigens to trigger kidney-specific autoimmunity;
3. Rare variants with alternative immunoglobulin subclass dominance (18) suggest that B cell class switching mechanisms may be more diverse than previously recognized.

### 3.3 Immunological features of nephrotic syndromes

The nephrotic syndrome can result from primary (idiopathic) nephropathy or a variety of secondary causes (19). The main pathological types of primary nephrotic syndrome include minimal change disease, focal segmental glomerulosclerosis, membranous nephropathy and membranoproliferative glomerulonephritis (20). While the secondary nephrotic syndrome is commonly seen in lupus nephritis, diabetic nephropathy and hepatitis B-related nephropathy, and each has its unique B cell immune mechanism. Early studies indicated that MCD was primarily caused by T cells, leading to podocyte foot process effacement and massive proteinuria. However, recent research has revealed that B cells play an auxiliary role in MCD pathology by secreting antibodies, providing antigenic stimulation signals to shape protective T cell responses, and regulating cytokines involved in T cell differentiation (21). Furthermore, the investigators found that autoantibodies produced by B cells are also key drivers in the immune pathogenesis of MN.

Notably, B cell dysregulation is also present in adult primary FSGS patients. Studies have shown significantly elevated proportions of CD27^+^IgD^-^IgG^+^ class-switched memory B cells in circulation, along with decreased CD38^+^IgG^+^ plasmablast proportions, although their specific pathogenic roles remain unclear (22). Glomerular basement membrane thickening and cellular proliferation in MsPGN patients are commonly associated with B cell-mediated immune complex deposition (23). In summary, B cells exhibit varying roles and levels of involvement across different pathological types (as shown in **Table 1**), suggesting the need for individualized therapeutic strategies based on pathological classification, with prognoses varying accordingly.

**Table 1:** B cell subset alterations in different types of nephrotic syndrome.

Types of disease	Reference	N	Study design	Discovery
**MCD**	Yokoyama H, et al. 1985 (227)	32	Retrospective	**Increase:** during disease activity,B lymphocytes (surface Ig-positive cells) and their subsets, B gamma, B mu, and B epsilon. **Decrease:** On treatment, B lymphocyte subsets tend to return to normal levels, accompanied by reductions in T3 and T4.
	Papakrivopoulou E et al. 2016 (179)	15	Prospective	When the circulating CD19 count is less than 100/μl, MCD is more likely to relapse.
	Oniszczuk J, et al. 2022 (56)	22	Cohort studies	**Increase:** The median percentage of plasmablasts increased in recurrent MCD patients. The increase of B cell activating factor (BAFF) production was significantly correlated with the percentage of plasmablasts.
	Cara-Fuentes G, et al. 2014 (228)	26	Observational	**Increase:** Levels of serum CD80, CD80 protein secretion and urinary CD80 excretion in recurrent MCD patients
	Tani Y, et al. 1985 (195)	15	Prospective RCT	**Increase:** Proportion of B lymphocyte subsets with surface immunoglobulin G (sIgG) in MCD, MN, IgA nephropathy, and MCGN
**MN**	Rosenzwajg, et al., 2017 (68)	25	Prospective-RCT	**Increase:** naive B cells (IgD + CD27-), DN-B (IgD-CD27-) **Decrease:** CSM-B (IgD-CD27 +), NCSM-B (IgD + CD27 +)
	Zhang, et al. 2017 (229)	45	Prospective-RCT	**Increase:** naive B cells (IgD + CD27-), plasma cells (CD138 + CD19 +) **Decrease:** NCSM-B (IgD + CD27 +)
	Cantarelli, et al. 2020 (5)	30	Prospective-RCT	**Increase:** plasma cells (CD19 + CD38^hi^CD27^hi^CD138 +) PLA2R-specific IgG-producing plasmablasts were recognized after *in vitro* expansion.
	Zhu, et al. 2023 (230)	28	Prospective-RCT	**PLA2R-MN:** circulating PLA2R antigen-specific memory B cells were increased
	Wang, et al. 2024 (231)	31	Prospective-RCT	**MN:** NCSM-B (IgD + CD27 +) and MZ-B (CD27 + IgD + IgM +) were decreased; plasmablasts (IgD-CD27^hi^CD38^hi^) were increased. **MN-New:** CSM-B (IgD-CD27^int^), NCSM-B and MZ-B were decreased; naive B cells (IgD +) and plasmablasts were increased. **MN-Rel:** NCSM-B were decreased, plasmablasts were increased.
	Deng, et al. 2024 (232)	26	Prospective-RCT	**Increase:** naive B cells (IgD + CD27-), plasma cells (CD38 + CD138 +) **Decrease:** CSM-B (IgD-CD27 +)
**FSGS**	Liu J, et al. 2021 (22)	16	Observational	**Increase:** CD27 + IgD- class-switched memory B cells 、CD27 + IgD-IgG + class-switched memory B cells (P < 0.0001), CD27hiCD38hi plasmablasts 、CD138 + plasma cells. **Decrease:** CD38 + IgG + plasmablast and serum IgG levels.
	S. Anis, et al. 2018 (12)	–	Abstract	**Increase:** CD5+B cells The high expression of CD5+B cell subsets may be related to the severity of the disease.
	Dan Hu Q, et al. 2023 (233)	10	Prospective	B cell translocator 2 (Btg2) can promote FSGS induced by adriamycin (ADR).
**Adult NS**	Casiraghi F, et al. 2023 (234)	18	Retrospective	**Increase:** CD19+CD20+ B cells; The frequencies of total B cells and memory B cells increased; **Decrease:** regulatory T cells (Treg)
	Ye Q, et al. 2021 (235)	81	Prospective	**Increase:** Cd27-naive B cells, CD27-CD38 + B cells and plasmablast cells in SSNS patients **Decrease:** IgD + memory B cells, transitional B cells (CD24hi CD38hi) and CD27 + CD38 - B cells
**Pediatric NS**	Yang X, et al. 2019 (236)	94	Prospective case-control	**Increase:** Circulating total plasmablasts, plasma cells and mature naive B cells. **Decrease:** Levels of germinal center-like B cells and CD19 IgG B cells.
	Colucci M, et al. 2019 (150)	107	single-center retrospective	**Increase:** Transitional B cells and memory B cells, especially switching memory B cells; There was a significant inverse correlation between total B cell levels and serum protein levels at onset.
	Colucci M, et al. 2016 (35)	28	Retrospective	**After RTX treatment**, the reconstitution of memory B cells (IgM memory and switching memory subsets) predicted the risk of relapse.
	Al-Aubodah TA, et al. 2023 (38)	31	Observational longitudinal	Extra-follicular B cells, CD21low T-bet+ atypical B cells, and antibody secreting cells are a hallmark of childhood INS.
	Colucci M, et al, 2023 (182)	102	Multicenter observational	Higher circulating levels of memory B cells at the time of anti-CD20 infusion were significantly associated with a higher risk of relapse.
	Riganati M, et al. 2024 (237)	58	Prospective Cohort	**At onset:** circulating levels of total CD19 + and specific B-cell subsets (transitional, matural-naive, plasmablastic/plasmacytic, CD19 + CD27 +, unconverted, converted, and atypical memory B cells) are markedly elevated. **At relapse:** there was a marked increase in transitional CD19+CD27+ memory and unconverted memory B cells.
	Ling C, et al. 2021 (152)	69	Prospective Observational	The proportion of memory B cells in SSNS patients in relapse group was significantly increased, while the proportion of transitional B cells was significantly decreased. Transitional B cells, memory B cells and the ratio of transitional B cells to memory B cells (T/M) were significantly correlated with relapse.

NS, Nephrotic Syndrome; MCD, Minimal Change Disease; MN, Membranous Nephropathy; MN-New, Newly-onset Membranous Nephropathy; MN-Rel, Relapse of Membranous Nephropathy; BAFF, B cell Activating Factor; PLA2R, Phospholipase A2 Receptor; FSGS, Focal Segmental Glomerulosclerosis; T/M ratio, Transitional to Memory B cell ratio; IL-10, Interleukin 10; RCT, randomized controlled trial.

Bold word: Increase, increase of B lymphocyte subgroups; Decrease, reduction of B lymphocyte subgroups; PLA2R-MN, Membranous Nephropathy Associated with Phospholipase A2 Receptor MN, membranous nephropathy; MN-New, newly diagnosed membranous nephropathy; MN-Rel, recurrence of membranous nephropathy; After RTX treatment, rituximab treatment follows; At the onset, when nephrotic syndrome occurs; At relapse, when nephrotic syndrome recurs.

In addition to primary nephrotic syndrome, B lymphocyte dysregulation also plays a vital role in secondary nephrotic syndrome. Studies have found that naive B cells can activate naive B cells with many clonalities in LN (24). These clonal naive B cells can produce antibody-secreting cells and persist in circulation for several months, leading to persistent non-remission of the disease (25). In addition, circulating transitional B cells and plasmablast cells are enriched in LN patients (26). A small study of B cells in the peripheral blood of patients with DKD demonstrated elevated levels of CD19lo/^+^CD38^+^ plasma cells relative to healthy controls. CD19lo/^+^CD38^+^ B cell counts were positively correlated with albumin excretion rate and serum IgG level and negatively correlated with estimated glomerular filtration rate, implying that higher plasma cell frequencies were associated with worsening DKD (27). Secondary MN is common in patients with chronic hepatitis B virus infection. The seminal study of Lai et al. (28) demonstrated a fundamental difference in the pattern of B-cell responses between HBV-associated membranous nephropathy and primary membranous nephropathy.

### 3.4 Comparison between primary nephrotic syndrome and secondary nephrotic syndrome

B cells change through the system, we found that the secondary and primary nephrotic syndrome in B cell steady state change there are important differences (as shown in **Table 2**):

1. B cell activation mechanism: B cell activation in secondary nephrotic syndrome (e.g. LN, HBV-GN) is often driven by specific causes (e.g., autoantigens, viral antigens), while the mechanism of B cell activation in primary nephrotic syndrome is not fully understood and may be related to T cell dysfunction and cytokine imbalance (29, 30).
2. B cell subsets mode: secondary nephrotic syndrome is a common variety of B cell subsets imbalance, such as the DN in a LN the depletion of B cells, B cells), and primary nephrotic syndrome mainly for memory B cells and plasma cells change (27).
3. B cells in kidney tissues: In secondary nephrotic syndrome, common kidney B cells and plasma cells infiltrate, sometimes forming similar germinal center structures, and in primary nephrotic syndrome, relatively few B cells infiltrate in kidney tissues (31).
4. Autoantibodies spectrum: secondary nephrotic syndrome (e.g., LN, HBV-GN) of autoantibodies or abnormal antibodies against specific antigens, and primary nephrotic syndrome antibody, there may be resistance to podocyte proteins, but the specificity is low (28).

**Table 2:** Comparative B-cell dysregulation in primary vs secondary NS.

Feature	Primary Nephrotic Syndrome	Secondary Nephrotic Syndrome
Driving Mechanism	The mechanism of B cell activation is not fully understood and may relate to T cell dysfunction. Cytokine imbalance may play a role in the non-specific activation of B cells.	B cell activation is usually driven by specific causes (such as autoantigens or viral antigens). Direct infection or deposition of antigens in renal tissue can lead to local activation (30, 238).
Subgroup Pattern	Mainly characterized by changes in memory B cells (CD19+ CD27+) and plasma cells, with relatively small changes in the proportion of B cell subsets in peripheral blood (150).	There is an imbalance of multiple B cell subgroups. For example, in lupus nephritis, there is a decrease in B cells, an increase in the proportion of CD27+ memory B cells, and functional abnormalities (27).
Renal Infiltration	Relatively few B cells infiltrate the renal tissue; B cell infiltration is observed but does not form germinal center structures (76).	B cells and plasma cells are commonly infiltrated in the renal tissue, often forming structures similar to germinal centers, indicating active B cell responses.
Autoantibodies	Produces antibodies against podocyte proteins, but specificity is usually low; the spectrum of autoantibodies is relatively narrow and mainly consists of non-specific antibodies (57).	Produces specific or abnormal antibodies targeting specific antigens (e.g., anti-HBV antigens, anti-GAD65), with the formation of immune complexes exacerbating renal damage (28).
Clinical Impact	The impact on renal function damage and disease progression is unclear, with efficacy often dependent on the improvement of overall immune status (239).	Autoantibodies have a direct negative impact on disease progression and renal damage; B cell activation is associated with disease activity and renal damage.

## 4 Diversity and definition of B cell subsets

### 4.1 Molecular diversity of B lymphocyte subsets

**Figure 1:**
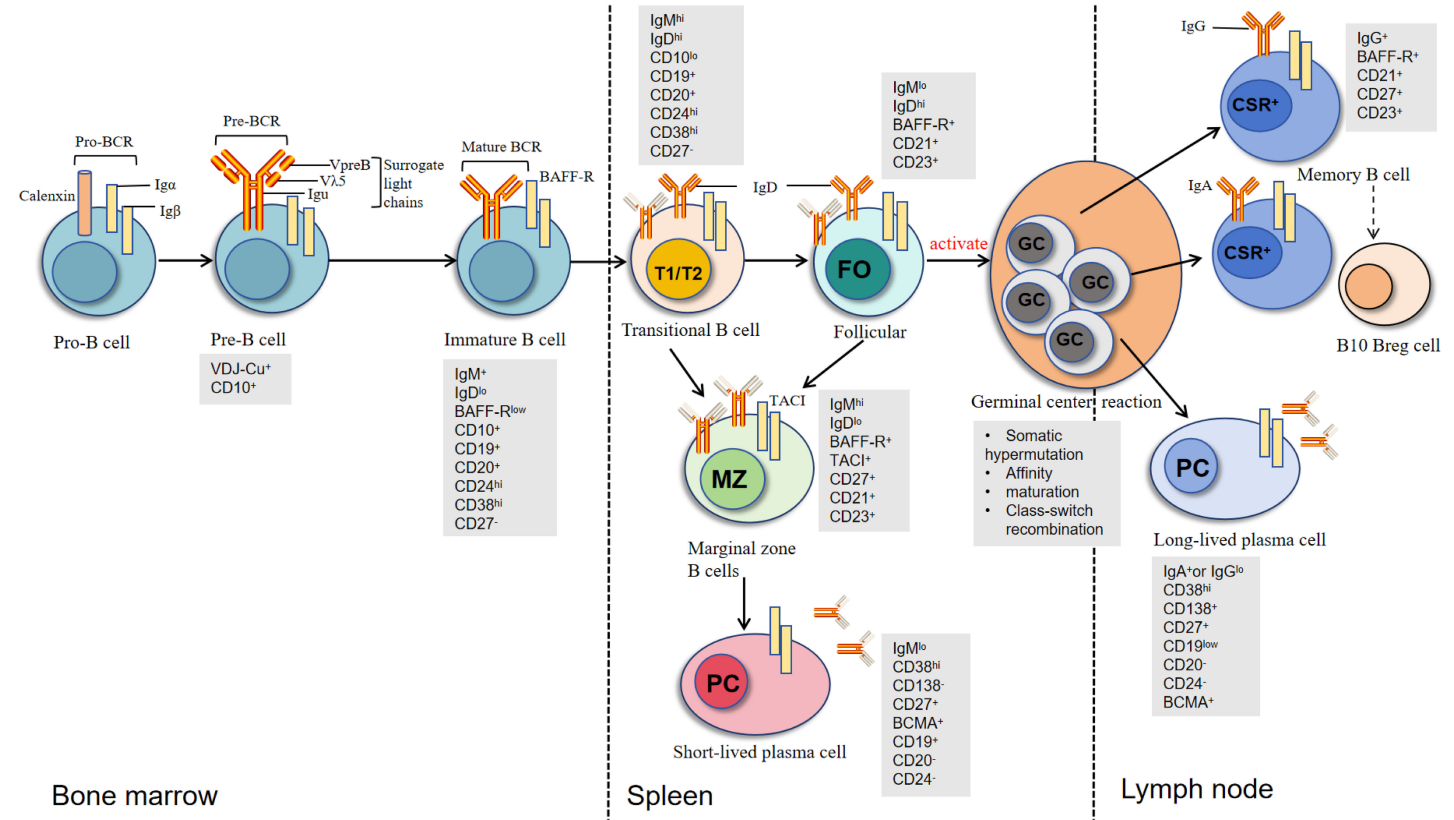
Development and differentiation of B cells across bone marrow, spleen, and lymph nodes. B cells develop sequentially from Pro-B cells to Pre-B cells and immature B cells in the bone marrow, marked by Pro-BCR/Pre-BCR expression and VDJ recombination. Immature B cells express IgM and undergo central tolerance screening before transitioning in the spleen through T1/T2 stages. They then differentiate into follicular B cells (FO), involved in adaptive immunity, or marginal zone B cells (MZ), which respond to blood-borne antigens. FO B cells further mature in lymph node germinal centers (GC) via somatic hypermutation and class switch recombination (CSR), producing memory B cells or long-lived plasma cells. Key markers (e.g., CD10, CD27) and immunoglobulin isotypes (IgM, IgD, IgG, IgA) are shown at each stage [modified from Oleinika K et al. (61)].

B lymphocyte development begins with pro-B cells in the bone marrow (as shown in **Figure 1**), where functional rearrangement of immunoglobulin genes, including heavy chain (IgH) and light chain (IgL) recombination, generates a B cell repertoire capable of recognizing diverse antigens. After expressing IgM(Immunoglobulin M) molecules, immature B cells migrate to the spleen through the transitional 1 (T1) stage and subsequently develop into transitional 2 (T2) B cells (32). T2 B cells can further differentiate into three major subsets: B1 cells, follicular B cells, and marginal zone (MZ) B cells. Among these, B1 cells can rapidly differentiate into short-lived plasma cells and secrete autoantibodies through T cell-independent mechanisms in nephrotic syndrome patients (33). Follicular B cells, as the predominant B cell subset, require CD4^+^ T cell help for activation and play a central role in the immune regulatory network of nephrotic syndrome (34).

### 4.2 B cell subsets and their roles in nephrotic syndrome

#### 4.2.1 Transitional B cells

Transitional B cells are immature B cells that migrate from the bone marrow to the spleen. They are typically characterized by the expression of high levels of CD24 and CD38 and further differentiate into various B cell subsets, including follicular and marginal zone B cells. Research indicates that transitional B cells are elevated in patients with SSNS and may serve as biomarkers for early disease detection (6, 35). Pathogenic mechanisms in NS: Immune tolerance defects: Reduced transitional B cell numbers disrupt peripheral immune tolerance mechanisms, allowing autoreactive B cells to escape negative selection (36). Relapse risk marker: The decreased ratio of transitional B cells to memory B cells is an independent risk factor for relapse in childhood steroid-sensitive nephrotic syndrome (37). Impaired regulatory function: Transitional B cells possess IL-10 secretion capacity; their reduction leads to insufficient anti-inflammatory regulation, promoting podocyte inflammatory injury (36).

#### 4.2.2 Non-class-switched memory B cells (IgM memory B cells)

These are a subtype of memory B cells that have not undergone class switch recombination and primarily produce IgM antibodies. They are essential for the initial immune response against previously encountered antigens and contribute to long-term immunity. In nephrotic syndrome, the persistence of non-class-switched memory B cells may indicate a sustained immune response and potential autoimmunity driving renal injury (32, 35). Pathogenic mechanisms in NS: Abnormal expansion and activation: Significantly increased in idiopathic nephrotic syndrome, serving as an important marker of disease activity (38). Autoantibody precursors: As precursors of antibody-secreting cells, they rapidly differentiate into plasma cells secreting IgM antibodies against podocyte antigens (36). Maintenance of chronic immune activation: Persistent IgM memory B cells maintain immune memory against podocyte-related antigens, driving disease relapse (37).

#### 4.2.3 Atypical B cells

Atypical B cells represent a heterogeneous population of functionally impaired B cells that expand during chronic inflammation and autoimmune conditions (39, 40). This population encompasses several distinct subsets with specific phenotypic and functional characteristics:

Age/autoimmune-associated B cells (ABCs) are characterized by high expression of T-bet (CD11^+^ T-bet^+^ CD21^low^) and represent a memory-like population that accumulates with age and in autoimmune diseases (41, 42). These cells are particularly prominent in systemic lupus erythematosus and other autoimmune nephritides, where they contribute to the production of pathogenic autoantibodies and chronic inflammatory responses (43, 44).

Double negative (DN) B cells (CD27⁻ IgD⁻) lack typical memory markers but exhibit activated phenotypes (45, 46). These cells are increased in patients with autoimmune diseases and can produce pathogenic autoantibodies, including anti-nuclear antibodies and anti-glomerular basement membrane antibodies (47). The expansion of DN B cells has been particularly well-documented in systemic lupus erythematosus and correlates with disease activity and organ damage.

Exhausted B cells (CD21^low^ CD38^low^) display reduced responsiveness to stimulation and impaired antibody production capacity (48, 49). Despite their “exhausted” phenotype, these cells can contribute to chronic inflammation through cytokine production and antigen presentation (50). This subset represents a tissue-like memory population that accumulates in sites of chronic inflammation and maintains immunological memory in pathological conditions.

In nephrotic syndrome, the expansion of these atypical B cell subsets has been associated with disease activity and treatment resistance, particularly in adult patients with minimal change disease and focal segmental glomerulosclerosis (46, 47). Their increased prevalence correlates with elevated levels of pro-inflammatory cytokines (IL-6, TNF-α, IFN-γ) and the production of autoreactive antibodies, contributing to the immunopathological mechanisms underlying proteinuria and glomerular damage (40, 42). Recent studies have also highlighted the potential of targeting these atypical B cell populations as a therapeutic strategy for steroid-resistant nephrotic syndrome (41, 47).

#### 4.2.4 Class-switched memory B cells

Class-switched memory B cells are differentiated B cells that have undergone class switch recombination to produce other immunoglobulin isotypes, primarily IgG or IgA. They are crucial for mounting robust immune responses upon re-exposure to pathogens. In FSGS, for instance, a significant increase in class-switched memory B cells has been observed and is associated with disease activity (22, 33). Pathogenic mechanisms in NS: Pathogenic antibody reservoir: In membranous nephropathy, class-switched memory B cells are an important source of pathogenic IgG4 antibodies such as anti-PLA2R and anti-THSD7A (51, 52). Immune complex formation: IgG antibodies produced bind to podocyte surface antigens, forming subepithelial immune complex deposits (53). Alternative complement pathway activation: IgG4 antibodies activate the lectin complement pathway through glycosylation alterations, leading to podocyte injury (51).

#### 4.2.5 Plasma cells

Plasma cells are terminally differentiated B cells that secrete large quantities of antibodies. They play an essential role in providing humoral immunity. In nephrotic syndrome, the increase in plasma cells correlates with elevated antibody levels and can reflect ongoing immune dysregulation. Moreover, abnormal plasma cell responses may lead to glomerular damage and worsen proteinuria (27, 28). Pathogenic mechanisms in NS: Continuous antibody secretion: Abnormally activated plasma cells continuously secrete large amounts of pathogenic antibodies, maintaining glomerular immune complex deposition (36). Local immune responses: Plasma cells infiltrating renal interstitium secrete antibodies locally, exacerbating local inflammatory responses and fibrosis (54). Treatment resistance mechanism: Long-lived plasma cells are independent of continuous antigen stimulation and represent an important cause of treatment resistance in some patients (36).

#### 4.2.6 Follicular B cells

Follicular B cells are a major subset of B cells located in the lymphoid follicles. They require T cell help for activation and play a pivotal role in generating robust antibody responses. In nephrotic syndrome, dysregulation of follicular B cells has been noted, which can impact overall immune function and contribute to disease pathology (34, 55). Pathogenic mechanisms in NS: Ectopic germinal center formation: Under chronic inflammatory stimulation, they can form ectopic germinal centers locally in the kidney, continuously producing autoantibodies (38). Participation in extrafollicular responses: Some follicular B cells can exit follicular structures and participate in extrafollicular immune responses, rapidly differentiating into antibody-secreting cells (38). Antigen presentation function: As antigen-presenting cells, they activate podocyte-specific T cells, amplifying autoimmune responses (42).

## 5 Clinical findings of major nephrotic syndromes

### 5.1 Minimal change disease

Recent clinical findings suggest that abnormal involvement of B cells may play a significant role in the pathogenesis and progression of MCD. Studies have shown that patients with MCD often exhibit elevated levels of B cells in peripheral blood, which may secrete cytokines (such as IL-4 and IL-13) that indirectly trigger abnormal T cell immune responses, consequently affecting glomerular permeability (55, 56). In 2022, Watts et al. (57) identified circulating autoantibodies against nephrin, a key podocyte slit membrane protein, for the first time in adults and children with MCD, and the levels of these autoantibodies correlated with disease activity (e.g., significantly higher positivity during flares than during remission). Furthermore, some MCD patients have been found to have anti-neurofascin antibodies, which correlate with disease activity, providing a new perspective on the underlying mechanisms of MCD that could aid early diagnosis in clinical settings (57, 58).

### 5.2 Focal segmental glomerulosclerosis

Studies on FSGS have indicated that an increase in the proportion of CD27^+^IgD^-^IgG^+^ switched memory B cells is associated with declines in renal function and increased 24-hour urine protein levels in patients (22, 59). This suggests that the activity level of memory B cells may reflect the severity of FSGS. In addition, Delville et al. (60), in their quest for potential biomarkers to predict FSGS recurrence, identified a panel of seven autoantibodies that could predict FSGS recurrence after transplantation with up to 92% accuracy in their cohort. In this population, elevated pre-transplant CD40 autoantibody, a transmembrane protein normally present on B cells, dendritic cells, and macrophages, had the best correlation with FSGS recurrence after transplantation. Moreover, notable infiltration of B cells has been observed in FSGS patients, which, in concert with T cells, may lead to podocyte injury and the development of proteinuria, deepening our understanding of B cell involvement in FSGS (61, 62).

### 5.3 Membranous nephropathy

In clinical studies of MN, PLA2R-related and THSD7A-related MN account for about 70%-80% and 1%-5% of primary MN patients, respectively, and are associated with disease activity. Elevated levels of these antibodies usually indicate disease recurrence (28, 63–65). Research indicates that approximately 40% of patients with MN are classified as PLA2R-negative MN. Among this group, exostosin 1/exostosin 2 (EXT1/EXT2) have been identified as the most common specific proteins, along with the discovery of neuro-epidermal growth factor-like 1 protein (NELL-1) and protocadherin 7 (PCDH7). Notably, MN associated with EXT1/EXT2 and NELL1-related malignant tumors may be linked to autoimmune diseases, such as systemic lupus erythematosus and mixed connective tissue disease. These autoimmune diseases are common in secondary MN; however, treatment for underlying secondary conditions (e.g., tumor removal) does not always correlate with improved outcomes in MN (66). Additionally, the proportion of memory B cells in MN patients significantly increases during periods of disease activity and decreases during remission, suggesting that these B cells may serve as biomarkers for disease activity (67, 68).

## 6 Pathogenic mechanisms of B lymphocyte subsets in different types of nephrotic syndrome

### 6.1 B Cell Immunoregulation in minimal change disease

**Figure 2:**
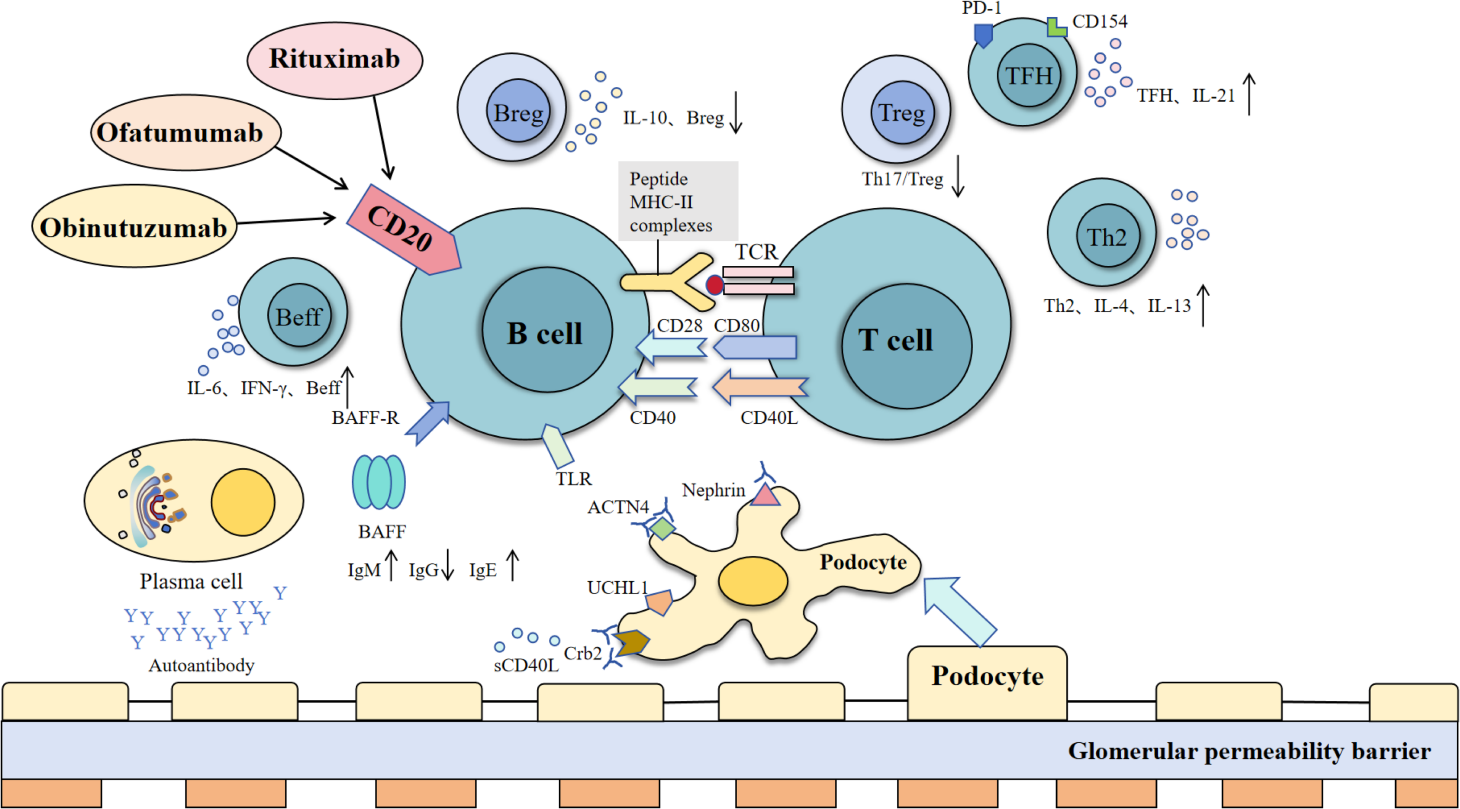
Interactions between B cells, T cells, and podocytes in immune regulation and glomerular barrier dysfunction. B cells interact with T cells via MHC-II and co-stimulatory signals (CD40-CD40L, CD28-CD80), supported by BAFF and TLR pathways, to promote activation, cytokine release (e.g., IL-6, IFN-γ), and antibody production. Regulatory B cells (Breg) suppress inflammation via IL-10, while effector B cells (Beff) amplify immune responses. Dysregulation of these processes leads to the production of autoantibodies, which target podocyte proteins (nephrin, ACTN4, Crb2), causing glomerular permeability barrier damage. T cell subsets, including TFH (IL-21), Treg, and Th2 (IL-4, IL-13), modulate B cell function, contributing to immune balance or pathology [modified from Liu et al. (243)].

Minimal change disease is the most common pathological type of nephrotic syndrome in children (over 1 year of age about 70%-90%), while the proportion in adults is low (15%) (69). MCD was first proposed by Shalhoub in 1974 as a T cell-mediated disorder (70). However, recent studies suggest that the interaction between B cells and T cells plays a crucial role in MCD pathogenesis (as shown in **Figure 2**).

**Figure 3:**
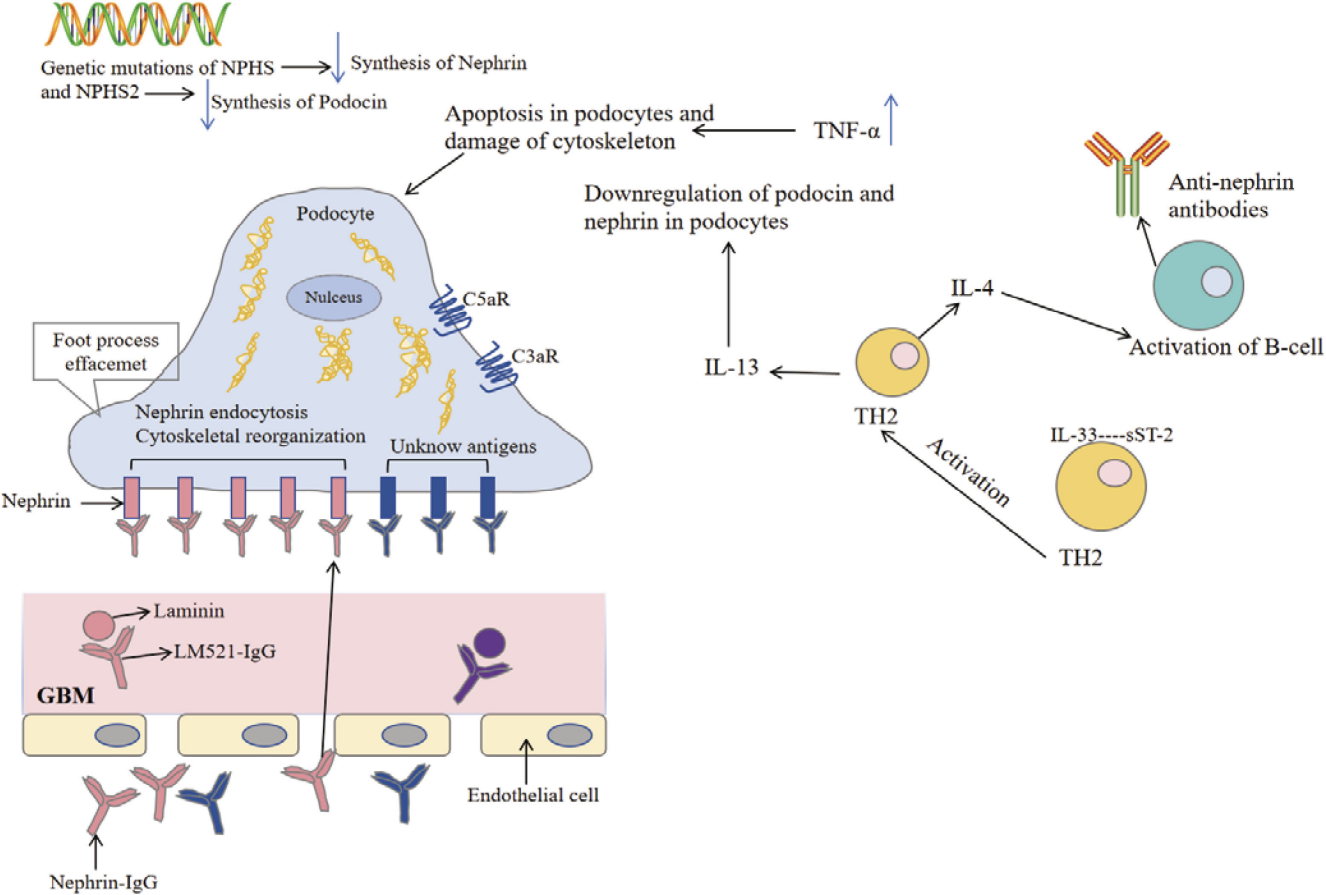
Pathophysiological mechanisms of minimal change disease. The pathogenesis of minimal change disease begins with genetic mutations in NPHS and NPHS2, leading to the synthesis of nephrin and podocin in the podocytes. The upregulation of tumor necrosis factor-α (TNF-α) induces apoptosis in podocytes and damages the cytoskeleton, resulting in foot process effacement and impairing the filtration barrier of the glomerulus. The endocytosis of antigens (such as nephrin) stimulates the activation of complement receptors (such as C5aR and C3aR), triggering an inflammatory response. During this process, the release of cytokines IL-4 and IL-13 activates TH2 cells, leading to the production of anti-nephrin antibodies by B cells, which further exacerbates podocyte damage. Additionally, these antibodies bind to the glomerular basement membrane, affecting endothelial cell function and exacerbating tissue injury. Collectively, these mechanisms contribute to the development of minimal change disease [modified from Cui Z et al. (57)].

The major mechanisms of B cells in minimal change disease include:

1. Abnormal immune regulation: it has been reported that the number and activation level of peripheral blood B cells in MCD patients are often increased. B cell activation can indirectly trigger abnormal T cell immune response by secreting cytokines (such as IL-4 and IL-13), and then affect glomerular permeability (56).
2. CD80 molecule mediating mechanism: in the patients with MCD of podocyte CD80 (B7 1) expression level, B cells through the secretion of cytokines, activate the cells express CD80, a study shows targeted CD80 treatment may reduce proteinuria (71, 72).
3. Autoantibodies impact: although MCD traditionally weak relations with autoantibodies, but some studies suggest can be detected according to individual patients cells themselves composition of low degree of autoantibodies, this may affect in membrane function and integrity (56).
4. Anti-nephrin autoantibodies: Recent studies have shown that anti-nephrin autoantibodies can be detected in some patients with minimal change disease. Recent studies have reported that anti-nephrin autoantibodies can specifically bind to podocytes (as shown in **Figure 3**), resulting in the accumulation of nephrin protein in the slit membrane, affecting its signal transduction and barrier function, and inducing proteinuria (58). Further experiments showed that the production of these antibodies could lead to immune complex deposition and activate local inflammatory responses, suggesting that B cell-mediated autoimmune mechanisms may exist in some cases of MCD (57, 58). The discovery of anti-nephrin antibody not only provides a new explanation for the pathogenesis of MCD, but also brings new possibilities for clinical diagnosis and precision treatment (such as immune intervention against antibodies).

### 6.2 The role of B cells in focal segmental glomerulosclerosis

Studies suggest that abnormal interactions between B lymphocytes and autoreactive T lymphocytes may play a crucial role in the pathogenesis of FSGS (73). Traditional views hold that B lymphocytes receive signaling stimulation in germinal centers (GC) by presenting antigens to T cells, subsequently differentiating into plasmablasts, antibody-secreting plasma cells, or long-lived memory B cells (59).

Current studies have shown that memory B cells participate in the development of primary FSGS through a variety of mechanisms:

1. Antibody-dependent mechanism: The increase of memory B cells in FSGS patients can promote the production of IgG and other immunoglobulins, form immune complexes that are deposited in the glomerulus, and induce cell damage and proteinuria (74).
2. Complement activation mechanism: IgG and IgM antibodies can cause the deposition of complement components (such as C3) by activating the classical pathway of complement or the alternative pathway of complement, and aggravate glomerular injury (75).
3. B cell infiltration and local effects: Focal B cell and T cell infiltration is obvious in glomeruli of FSGS and steroid-resistant nephropathy patients. Locally activated B cells can induce podocyte injury and promote the formation of proteinuria by secreting inflammatory cytokines and antibodies (61, 62). Furthermore, Benz et al. observed significant glomerular B cell infiltration in patients with FSGS and SRNS, providing additional support for the potential pathogenic role of B lymphocytes (76). Kim et al. discovered that locally activated B cells in glomeruli could induce podocyte injury and proteinuria in experimental mouse models, thereby exacerbating glomerular damage and promoting disease progression (61).
4. BAFF mediated effect: The expression of BAFF in podocytes and interstitial infiltrating cells is up-regulated in renal biopsy of some patients, and the high expression of BAFF is closely related to the decline of renal function (77). Additionally, in a study of 33 MCD or FSGS patients with kidney biopsies, BAFF expression was observed in podocytes and interstitial inflammatory infiltrates in 18.2% and 36.4% of biopsy samples, respectively. BAFF expression was associated with more rapid eGFR decline and overall reduced eGFR values (78, 79). Therefore, it is hypothesized that memory B cells may participate in the pathogenesis of FSGS and potentially serve as sensitive biomarkers for predicting the severity of primary FSGS. Although studies have revealed the role of B cell dysfunction in the pathogenesis of FSGS, further research is needed to identify the key pathogenic B cell subsets and their specific mechanisms in FSGS.

### 6.3 B cell-mediated mechanisms in membranous nephropathy

**Figure 4:**
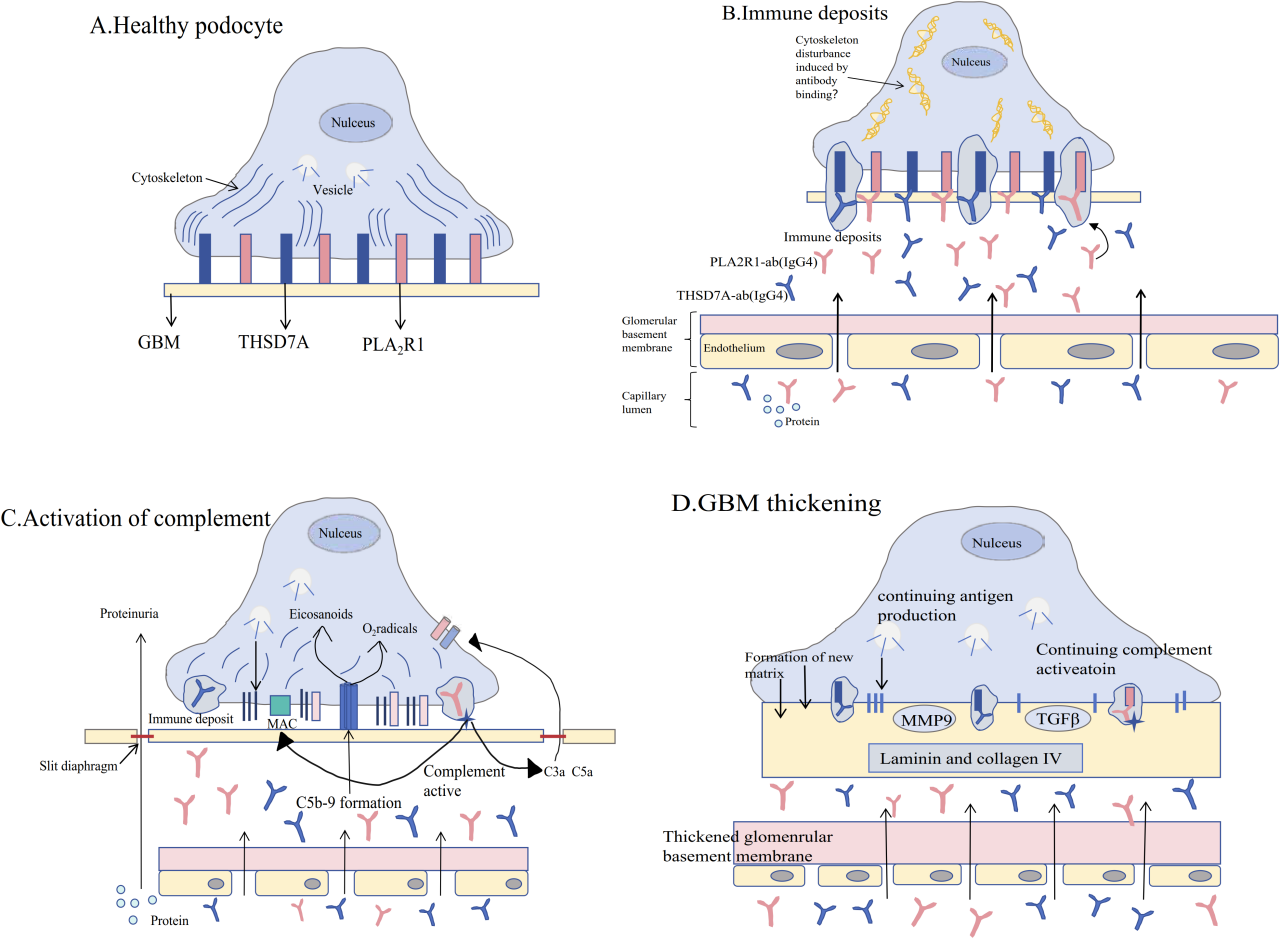
Pathophysiological mechanisms of membranous nephropathy. **(A)** The pathogenesis of membranous nephropathy (MN) [see Figure **(A)**] begins with the normal expression of endogenous antigens phospholipase A2 receptor 1 (PLA2R1) and thrombospondin type-1 domain-containing 7A (THSD7A) on podocytes in the glomerulus. Podocytes remain healthy in the absence of specific circulating antibodies. **(B)** When circulating antibodies bind to these antigens, immune deposits form, damaging the cytoskeletal integrity of podocytes and leading to the onset of proteinuria. **(C)** Immune deposits further activate the complement system, resulting in the formation of the C5b-9 membrane attack complex and promoting the activation of reactive oxygen species and eicosanoids, which disrupt the filtration barrier of the glomerulus and exacerbate proteinuria. **(D)** The persistent presence of pathogenic antibodies and chronic complement activation lead to thickening of the glomerular basement membrane (GBM), accompanied by podocyte effacement and glomerular capillary sclerosis, a process regulated by transforming growth factor-β (TGFβ) and matrix metalloproteinase-9 (MMP9) [modified from Ronco P et al. (81)].

B lymphocytes are central players in the pathogenesis of MN, directly influencing disease progression through the secretion of autoantibodies (such as PLA2R-Ab) and regulation of immune responses (as shown in **Figure 4**) (80). In contrast, PLA2R-Ab is merely a product of B cell function and cannot comprehensively reflect B cell activity status and their role in immune regulation. Current research has already established memory B cells as biomarkers for MN disease activity and predictors of relapse (68).

The main mechanisms include:

1. Antigen-antibody complex deposition: The fundamental pathogenesis of MN is that autoantibodies (such as anti-PLA2R and anti-THSD7A) produced by B cells target podocyte surface antigens, and specific IgG4 antibodies form immune complex deposition under the glomerular capillary loops (81).
2. Activation of the classical complement pathway: autologous IgG/immune complexes activate complement, especially C5b-9 (membrane attack complex), which is locally formed in podocytes, and its products can directly damage podocytes, resulting in the loss of slit membrane proteins and proteinuria (82).
3. B cell subsets and disease activity: peripheral plasma cells and memory B cells in MN patients were significantly increased in the active stage of disease, and were correlated with antigen and antibody titers. Anti-cd20 monoclonal antibody can effectively eliminate pathogenic B cells, induce a decrease in antibody titer and clinical remission (67). The above mechanisms suggest that pathogenic autoantibodies secreted by B cells are the core pathological basis of MN, while complement-dependent podocyte injury and local inflammatory responses are the direct drivers of pathological changes.

### 6.4 Emerging pathological mechanisms and their interactions with B cells in membranous nephropathy

#### 6.4.1 Recent studies have identified several novel pathological mechanisms in MN that interact with B cell-mediated immune responses, providing new insights into disease complexity and potential therapeutic targets.

##### 6.4.1.1 Sirtuin expression and regulation

Sirtuin family proteins, particularly SIRT6, play crucial regulatory roles in MN pathogenesis. SIRT6 protects against podocyte injury by blocking the renin-angiotensin system through inhibition of the Wnt1/β-catenin pathway (83). Recent breakthrough research demonstrates that SIRT6 exerts dual protective mechanisms: directly deacetylating β-catenin to reduce nuclear translocation while simultaneously suppressing local RAS components including angiotensinogen and ACE expression in podocytes (83).

Importantly, sirtuins also regulate B cell activation and antibody production processes. SIRT1 deficiency can lead to excessive B cell activation and increased autoantibody production, which may explain the abnormal B cell responses observed in some MN patients (83). Enhanced understanding reveals that SIRT6 specifically modulates B cell class switching and memory formation, with deficiency promoting pathogenic IgG4 subclass production characteristic of MN pathogenesis (83). This finding suggests that targeting sirtuin pathways could provide dual benefits by protecting podocytes while modulating aberrant B cell immunity.

##### 6.4.1.2 Renin-angiotensin system activation

RAS activation not only directly damages glomerular structures but also influences immune cell function. The intrarenal RAS system plays a critical role in MN progression through complex interactions with immune mechanisms (84). Recent evidence reveals that RAS activation creates a proinflammatory microenvironment that specifically promotes autoreactive B cell activation and anti-PLA2R antibody production (83). Angiotensin II promotes B cell differentiation toward plasma cells and enhances antibody production capacity. Advanced pathophysiological mechanisms include: Angiotensin II directly stimulating B cell differentiation toward plasma cells through AT1 receptor signaling, RAS activation enhancing BAFF (B cell activating factor) expression in renal tissues, and the ACE2/Ang(1-7)/Mas axis imbalance reducing anti-inflammatory regulatory B cell function (83). Furthermore, imbalance of the ACE2/Ang(1-7)/Mas axis may lead to reduced anti-inflammatory regulatory B cell function, further exacerbating immune-mediated injury in MN.

##### 6.4.1.3 Wnt1/β-catenin signaling pathway

Abnormal activation of the Wnt1/β-catenin pathway plays a key role in MN progression. This pathway not only affects podocyte survival and function but also regulates B cell differentiation and memory formation (85). Enhanced mechanistic understanding reveals that excessive β-catenin activation promotes memory B cell transformation into long-lived plasma cells, leading to sustained anti-PLA2R antibody production, while Wnt signaling dysfunction impairs podocyte autophagy and enhances susceptibility to immune complex-mediated injury (86). Excessive β-catenin activation can promote memory B cell transformation into long-lived plasma cells, leading to sustained antibody production and immune complex deposition. Moshen granule has been shown to ameliorate MN by blocking intrarenal RAS signaling via the Wnt1/β-catenin pathway, demonstrating the interconnection between RAS, Wnt signaling, and immune responses (87). Additionally, Lactobacillus species ameliorate MN through modulation of this pathway, highlighting the gut-kidney axis involvement (86). This represents the first direct mechanistic link between gut microbiota metabolites and MN pathogenesis through tryptophan-produced indole metabolites that inhibit the aryl hydrocarbon receptor (AhR) pathway, which subsequently modulates Wnt1/β-catenin signaling (86).

##### 6.4.1.4 Immunoglobulin A-κ deposition mechanisms

Recent findings reveal that IgA-κ light chain deposition may occur in some MN patients, representing a fundamentally distinct pathological pattern from classical IgG4-dominant deposition. Unlike the typical IgG4 subclass predominance seen in most MN cases, this IgA-κ deposition pattern suggests alternative B cell activation pathways and distinct immunological mechanisms (88). Groundbreaking discovery reveals that some patients presenting with membranous nephropathy-like patterns may actually have fibrillary glomerulonephritis with IgA-κ light chain deposition rather than classical IgG4-dominant deposition (89).

This IgA-κ deposition appears to be associated with specific B cell clonal expansion that produces aberrant IgA antibodies, which subsequently form immune complexes in glomeruli (90). The shift from IgG4 to IgA production indicates different B cell subset activation and class-switching mechanisms, potentially involving distinct cytokine environments and T helper cell responses compared to classical MN (91). Key pathophysiological insights include: unlike typical IgG4 subclass predominance, IgA-κ deposition patterns suggest alternative B cell activation pathways involving distinct immunological mechanisms, and immunoelectron microscopy reveals unique ultrastructural features distinguishing these cases from classical membranous nephropathy (89). This clonal expansion of IgA-producing B cells may represent a unique pathogenic pathway that bypasses the conventional IgG4-mediated immune complex formation (92).

The identification of this distinct deposition pattern has significant therapeutic implications. Patients with IgA-κ deposition may respond differently to standard immunosuppressive therapies designed for IgG4-dominant MN, potentially requiring B cell-targeted approaches that specifically address IgA-producing clones (92).

##### 6.4.1.5 Gut Microbiota dysbiosis and the gut-kidney axis

Gut microbiota dysbiosis significantly influences MN development and progression through the gut-kidney axis. Two-sample Mendelian randomization studies have established causal relationships between specific gut microbiota compositions and kidney diseases, providing robust evidence for the gut-kidney axis (93). This approach eliminates confounding factors and reverse causation, establishing microbiota as a genuine causal factor rather than merely an association (93). Systematic analysis reveals specific alterations in gut microbiota composition in MN patients, with expansion of Proteobacteria and depletion of Lachnospira being critical features (94). Comprehensive microbiota characterization includes: expansion of Proteobacteria phylum particularly pathogenic Enterobacteriaceae, critical depletion of beneficial Lachnospira species and other short-chain fatty acid (SCFA) producers, reduced microbial diversity indices correlating with disease activity and proteinuria severity, and distinct microbiota signatures distinguishing MN from other glomerular diseases (94). Altered intestinal microbial flora and metabolism in patients with idiopathic MN demonstrate significant correlations with disease activity (95).

Advanced mechanistic networks demonstrate that altered intestinal microbial flora and metabolism in patients with idiopathic MN show significant correlations with disease activity (95). Microbiota imbalance can compromise intestinal barrier function, increasing translocation of bacterial antigens and toxins that activate systemic immunity. This chronic immune activation state may promote autoreactive B cell generation and molecular mimicry of podocyte antigens. Mechanistic pathways include: microbiota imbalance compromising intestinal barrier function and increasing translocation of bacterial antigens and endotoxins, chronic low-grade endotoxemia activating systemic immunity and promoting autoreactive B cell generation through molecular mimicry, certain bacterial metabolites particularly SCFAs possessing regulatory B cell (Breg) functions with microbiota dysbiosis disrupting this regulatory balance, and tryptophan metabolism dysfunction reducing production of anti-inflammatory indole derivatives (95). Additionally, certain bacterial metabolites such as short-chain fatty acids possess B cell regulatory functions, and microbiota dysbiosis may disrupt this regulatory balance.

##### 6.4.1.6 Interconnected mechanistic networks

Novel environmental mechanism has identified the aryl hydrocarbon receptor (AhR) pathway as a critical mediator linking environmental toxins to MN pathogenesis (86). This pathway serves as a molecular sensor for environmental pollutants and dietary toxins, directly influencing immune cell function and glomerular injury with advanced mechanistic understanding including: environmental toxins activating AhR signaling and promoting proinflammatory cytokine production and B cell activation, AhR activation specifically enhancing Th17 cell differentiation while suppressing regulatory T cell function, the pathway directly influencing podocyte metabolism and oxidative stress responses, and beneficial microbiota metabolites (indole derivatives) antagonizing AhR activation (86).

These emerging mechanisms do not operate independently but form complex interaction networks. For example, RAS activation can promote Wnt pathway activation, sirtuin protein deficiency can exacerbate gut microbiota dysbiosis, while microbiota metabolites can regulate sirtuin expression (96). This multi-mechanistic interaction explains the complexity of MN pathological processes and provides theoretical basis for multi-target combination therapeutic strategies targeting both traditional immune pathways and these newly identified mechanisms.

###### 6.4.1.6.1 Multi-level integration includes:

1. Environmental triggers (toxins, dysbiosis) → AhR pathway activation → Wnt/β-catenin signaling → podocyte injury;
2. RAS activation → SIRT6 suppression → enhanced Wnt signaling → B cell activation → autoantibody production;
3. Microbiota dysbiosis → reduced SCFA production → impaired Breg function → enhanced autoreactive B cell responses (86).

#### 6.4.2 Enhanced integration with B cell biology

Revolutionary B cell mechanistic insights:

##### 6.4.2.1 Environmental-B cell interactions

1. Microbiota-derived metabolites directly regulate B cell class switching and memory formation (86, 95);
2. Environmental toxins through AhR signaling promote pathogenic B cell activation while suppressing regulatory functions (86);
3. The gut-kidney axis represents a novel pathway for environmental antigens to trigger kidney-specific autoimmunity (93, 94).

##### 6.4.2.2 Metabolic-immune integration

1. Sirtuin-mediated metabolic regulation directly influences B cell energetics and antibody production capacity (86);
2. Wnt/β-catenin signaling serves as a metabolic checkpoint controlling B cell differentiation and memory formation (86);
3. These pathways represent novel therapeutic targets for modulating B cell responses in MN (86);

##### 6.4.2.3 Clinical implications

1. Microbiota profiling may serve as a biomarker for treatment response and disease progression (94, 95);
2. Combination therapies targeting multiple pathways may provide superior outcomes compared to single-agent approaches (86);
3. Precision medicine approaches based on individual mechanistic profiles may revolutionize MN treatment (89, 93).

### 6.5 B cell immune network in membranoproliferative glomerulonephritis

B lymphocytes play crucial roles through multiple pathways in the pathogenesis of MPGN. Primarily, immunoglobulins (such as IgG and IgM) secreted by B lymphocytes form immune complexes with antigens, which deposit in the glomerular basement membrane and mesangial areas. These complexes activate the complement system, triggering inflammatory responses and glomerular injury—a core mechanism in immune complex-mediated MPGN (97). Additionally, antibodies produced by B lymphocytes can exacerbate inflammatory responses through the classical complement pathway. Even in complement-mediated MPGN, complement overactivation may be indirectly influenced by B cell function. In some MPGN patients with chronic viral infections [such as Hepatitis C Virus (HCV) and Hepatitis B Virus (HBV)], B cells maintain chronic immune activation by producing antiviral antibodies, thereby accelerating disease progression (64).

Furthermore, abnormal B lymphocyte proliferation and aggregation may closely correlate with MPGN’s pathological changes and disease severity, particularly in systemic lupus erythematosus-associated MPGN (98). B cells also interact with other immune cells (such as T cells and macrophages) through antigen presentation and cytokine secretion, further aggravating glomerular inflammation and injury (99). B cell-targeted therapy has demonstrated good efficacy in MPGN patients by selectively depleting B cells, reducing antibody and immune complex formation, and suppressing inflammatory responses, thereby improving proteinuria and renal function. In 2012, Dillon et al. treated 6 MPGN patients with rituximab, achieving complete remission in 5 cases (100). Another single-center retrospective study included 2 MPGN patients. One patient achieved complete remission after one or two doses of rituximab (375 mg/m²), while the other achieved partial remission before developing crescentic rapidly progressive glomerulonephritis requiring dialysis. However, this patient discontinued dialysis after 5 months and achieved complete remission at 14 months (101). Moreover, RTX showed remarkable efficacy in HCV-related MPGN patients, with proteinuria decreasing from 8.2g/24h to 0.62g/24h within one year, while serum creatinine and liver function remained stable (102, 103). These findings indicate that B lymphocytes play a vital role in MPGN pathogenesis and progression while serving as important therapeutic targets.

### 6.6 B-cell abnormalities in lupus nephritis

With lupus nephritis is abnormal B cells in secondary nephrotic syndrome is the most typical representative, its characteristic changes include:

1. Amplification of Double Negative B Cells: In patients with LN, there is a significant amplification of CD19^+^CD27^-^IgD^-^”double negative B cells,” which produce high-affinity autoantibodies correlated with the severity of renal injury (104).
2. B Cell Infiltration in Renal Tissue: Over 50% of LN patients exhibit CD20+ B cell infiltration in renal tissue, forming structures resembling lymphoid follicles (31).
3. Abnormal TLR Signaling Pathway: In LN patients, the TLR7/9 signaling pathway is abnormally activated in B cells, leading to persistent activation of autoreactive B cells and the production of autoantibodies (105).

### 6.7 Immunopathological role of B cells in diabetic nephropathy

Although diabetic nephropathy has traditionally been considered primarily a metabolic disease, recent studies have revealed the important role of B-cell-mediated inflammatory and immune mechanisms in its pathogenesis:

1. B Cell Trafficking in Diabetic Nephropathy: The upregulation of B cell chemokine CXCL13 in renal tissues of diabetic nephropathy (DN) patients provides a microenvironmental basis for B cell involvement in the disease (106). This feature is not commonly seen in primary nephrotic syndrome.
2. Memory B Cell Infiltration: Significant infiltration of memory B cells has been observed in the glomeruli of DKD models, particularly in the NOD mouse model (27). In patients with type 2 diabetes, increased CD20^+^ B cells in the renal interstitium correlate with proteinuria levels.
3. Autoantibody Production and Damage: Autoantibodies against glomerular proteins, such as anti-GAD65 and anti-oxidized LDL (27), are present in DN patients and deposited in glomeruli, exacerbating kidney injury through immune complex formation (107).

### 6.8 Unique manifestations of B cells in hepatitis B-related nephropathy

B-cell abnormalities in HBV-related nephropathy (predominantly membranous nephropathy) have unique features:

1. Mechanisms of *In Situ* Immune Complex Deposition: Studies indicate that HBV-DNA is present in the glomerulus, potentially inducing immune complex formation through direct infection of glomerular cells or expression of viral antigens (108). Three primary mechanisms of immune complex formation in HBV-associated glomerulonephritis (HBV-GN) include circulating immune complex deposition, *in situ* immune complex formation, and direct viral infection of renal cells (30).
2. Specific Antibody Responses in HBV-GN: In HBV-associated membranous nephropathy (HBV-MN), B cells predominantly produce specific antibodies against viral antigens such as HBsAg and HBeAg (28). This contrasts with primary membranous nephropathy, where anti-PLA2R antibodies are the primary antibodies, informing different treatment strategies for the two conditions.
3. Activation of B Cells in Renal Tissues: Single-cell sequencing analysis of renal biopsy samples from HBV-MN patients reveals that infiltrating B cells in the kidneys exhibit higher levels of activation markers (HLA-DR and CD86) and specifically produce antibodies against HBV antigens, indicating a distinct immune response compared to primary membranous nephropathy (109).

## 7 Treatment of major nephrotic syndromes

### 7.1 Minimal change disease

Minimal Change Disease is the most common type of nephrotic syndrome in children, with its primary characteristic being an excellent response to corticosteroid therapy. Prednisone is the standard first-line treatment, and approximately 80-90% of children achieve remission within 4–6 weeks of starting therapy (110, 111). For patients who are steroid-resistant or experience frequent relapses, alternative treatments such as tacrolimus and mycophenolate mofetil (MMF) have been shown to be effective. Research suggests that these immunosuppressive agents can induce and maintain remission in steroid-resistant cases (112, 113).

In resistant cases of MCD, tacrolimus has proven particularly beneficial, with many patients achieving remission after transitioning to this treatment (113). Additionally, the efficacy of rituximab in adult patients with MCD has been confirmed by multiple studies. A systematic review and meta-analysis indicated that the overall remission rate for MCD patients treated with rituximab was 80.3% (95% CI, 68.5–88.5%) (114). Another retrospective study involving 21 adult patients found that all patients achieved clinical remission after treatment, with 19 (90.48%) attaining complete remission and 2 (9.52%) achieving partial remission (115). Moreover, RTX shows great promise in the treatment of recurrent MCD patients, especially in hormone-dependent or frequent recurrent patients. The 2014 NEMO study reported reduced relapse rates in patients treated with RTX (116). The KDIGO guidelines recommend broader use of RTX, especially in steroid-dependent and frequently relapsing cases (117).

Emerging therapeutic strategies have shown promise in addressing the pathophysiological basis of MCD. Recent studies demonstrate that natural triterpenoid compounds, particularly those derived from Poria cocos, offer significant nephroprotective effects through multiple mechanisms including inhibition of RAS, TGF-β1/Smad3, and Wnt/β-catenin pathways (118). The integration of gut microbiota modulation strategies presents novel therapeutic opportunities, particularly through restoring the balance of beneficial bacteria such as Lachnospira and reducing harmful bacteria like Shigella and Streptococcus, which have been identified as dysregulated in MCD patients (119).These microbiota-targeted interventions may complement traditional immunosuppressive therapies by addressing underlying immune dysregulation at the gut-kidney axis level.

### 7.2 Focal segmental glomerulosclerosis

FSGS presents significant challenges due to its heterogeneous nature and varying treatment responses. Corticosteroids remain the preferred treatment, though their efficacy can vary among patients. While corticosteroids are often used as a first-line treatment, the response rate is considerably lower in adults, necessitating additional treatment options (120).

The combination of corticosteroids with tacrolimus and MMF has shown promise for patients resistant to glucocorticoids. A retrospective study in children with nephrotic syndrome found that this combination achieved a response rate of 75% in refractory cases (121). Furthermore, in patients resistant or dependent on cyclosporine A (CsA), tacrolimus combined with corticosteroids has been effective in inducing sustained reductions in urinary protein (122). Notably, a study involving 25 patients with FSGS who were resistant or dependent on CsA reported that 100% achieved remission after treatment with tacrolimus (122).

Emerging therapies for FSGS, such as sparsentan, have demonstrated effectiveness in clinical trials, particularly in reducing proteinuria and outperforming traditional ACE inhibitors (123). Additionally, anti-CD20 monoclonal antibodies (like rituximab) have shown significant efficacy in resistant FSGS cases, with remission rates exceeding 50% (124, 125). Innovative agents like Barleriside A are also being investigated, acting as aryl hydrocarbon receptor antagonists to ameliorate oxidative stress and inflammation, representing a potential new therapeutic option for FSGS (119).

Advanced therapeutic approaches are emerging based on novel mechanistic insights. Aryl hydrocarbon receptor (AhR) pathway modulation represents a promising therapeutic target, with natural compounds like BSA demonstrating high-affinity AhR antagonist properties that eliminate oxidative stress and inflammation through regulation of IκB/NF-κB and Keap1/Nrf2 pathways (126). Furthermore, comprehensive podocyte protection strategies utilizing traditional Chinese medicines have shown remarkable efficacy through multiple mechanisms including inhibition of JNK/ERK signaling pathways, restoration of podocyte actin cytoskeleton, reduction of C5b-9 complex deposition, and enhancement of Nrf2/HO-1 pathways (127). The application of matrix metalloproteinase (MMP) inhibition strategies represents another innovative approach, targeting the critical role of MMPs in renal fibrosis and matrix remodeling processes that characterize FSGS progression (118).

### 7.3 Membranous nephropathy

Membranous nephropathy is a major cause of nephrotic syndrome in adults, necessitating a tailored treatment approach focused on immunoregulation. Corticosteroids remain the cornerstone of therapy for high-risk patients and those with severe symptoms, often combined with other immunosuppressants to enhance efficacy. Recent studies indicate that the combination of glucocorticoids with tacrolimus and mycophenolate mofetil significantly improves outcomes, especially in patients with underlying conditions like type 2 diabetes mellitus (128).

Revolutionary combination therapy approaches have emerged for complex cases. A groundbreaking retrospective study demonstrates that low-dose multi-target therapy (prednisone 10mg/d + tacrolimus 0.05mg/kg/d + mycophenolate mofetil 1g/d) significantly outperforms cyclophosphamide-based regimens in pMN patients with concurrent type 2 diabetes mellitus. This novel approach achieved superior proteinuria reduction (-4800.48 ± 3002.65 vs -1663.32 ± 4113.98 mg/24h at 2 months, P-BH=0.045) while maintaining better glycemic control (128). Such precision therapy approaches represent a paradigm shift toward individualized treatment strategies that consider comorbidity profiles.

Calcineurin inhibitors, such as tacrolimus and cyclosporine, have effectively reduced proteinuria and improved renal function (129, 130). Cyclophosphamide may also be employed in certain cases of immune-mediated MN (131). Clinical trials have demonstrated RTX’s superior efficacy in MN compared to traditional therapies. The GEMRITUX trial (132) indicated a remission rate of 64.9% for RTX patients after 17 months, compared to 34.2% for those receiving supportive care. The MENTOR trial (133) further established RTX’s effectiveness, with 62% of the RTX group achieving complete or partial remission at two years, versus 20% in the cyclosporine group. RTX’s efficacy involves dismantling autoreactive B lymphocyte clones that produce pathogenic anti-PLA2R antibodies (134), with a meta-analysis confirming a total remission rate of 67% and an average proteinuria reduction of 4.90 grams/day in MN patients. Additionally, B-cell targeted therapies, including rituximab and belimumab, have shown promising outcomes in enhancing patient prognosis (135).

Innovative integrated approaches combining low-dose cyclophosphamide with traditional Chinese medicine formulations represent a breakthrough in personalized medicine. The Shulifenxiao formula combined with low-dose cyclophosphamide has demonstrated excellent efficacy and safety across different risk stratifications of primary MN patients, offering a novel Chinese-Western integrated therapeutic paradigm (136).

Comprehensive network Meta-analysis of 31 randomized controlled trials involving 2,195 patients has established the superior efficacy of multiple Chinese medicine formulations. Fifteen distinct Chinese medicine preparations including Wuweizi capsules (WZC), Leigongteng multiglycoside tablets (LGT), Shengmai injection (SMI), Shenshen Kang capsules (SFC), Bailing capsules (BLC), Dihuangye total glycoside capsules (DHT), Qingre Qushi granules (QRG), Shenyan Kangfu tablets (SYT), Qiqi Yishen capsules (QQC), Huangqi injection (HBT), Renkang injection (SKI), and Huangkui capsules (HKC) have demonstrated superior outcomes in improving 24-hour proteinuria, serum albumin, creatinine, total cholesterol, and triglycerides compared to conventional therapy alone (137).

CD4+ T cell subset modulation represents another innovative therapeutic approach. Jianpi Qushi Heluo Formula (JQHF) has demonstrated remarkable efficacy in correcting CD4+ T cell subset imbalances characteristic of pMN patients (elevated Th1/Th17 cells, reduced Th2/Treg cells) while significantly reducing CXCL10 levels, which correlate with 24-hour proteinuria levels (138). This targeted immune regulation approach addresses the fundamental pathophysiological mechanisms underlying MN progression.

In addition to conventional therapies, recent research has explored the potential of Chinese herbal medicine to inhibit podocyte damage as a therapeutic strategy for MN. This approach may serve as a valuable adjunct to standard treatments (139). The traditional use of Poria cocos has also been examined for its nephroprotective properties, with studies focusing on its triterpenoid components and their pharmacological effects (118).

Advanced podocyte protection strategies utilizing traditional Chinese medicines represent a comprehensive therapeutic approach addressing multiple injury mechanisms. These strategies target complement system activation (C5b-9 membrane attack complex, C3a/C3aR pathways), pyroptosis and autophagy dysregulation (NLRP3 inflammasome activation, mTOR pathway abnormalities), and oxidative stress (increased ROS production, NADPH oxidase upregulation). Specific interventions include Astragalus IV for JNK/ERK pathway inhibition and podocyte actin cytoskeleton restoration, Triptolide for C5b-9 complex deposition reduction and p38MAPK pathway inhibition, Curcumin for PI3K/AKT/mTOR pathway inhibition and Nrf2/HO-1 pathway enhancement, Zhenwu decoction for NF-κB pathway and NLRP3 inflammasome inhibition, and Sanqi oral solution for Nrf2/HO-1 pathway activation and NF-κB pathway inhibition (127).

### 7.4 Mechanistic basis of B cell-targeted therapies

B cell-targeted therapies, particularly anti-CD20 monoclonal antibodies, have revolutionized nephrotic syndrome management, highlighting the critical role of B cells in disease pathogenesis and offering alternative options for patients with limited therapies. Anti-CD20 monoclonal antibodies like RTX exert therapeutic effects through various mechanisms. In addition to direct B cell depletion, RTX modulates T cell function by depleting a small percentage of circulating T lymphocytes (140), disrupting autoreactive loops between B and T cells, thereby reducing inflammation and tissue damage (21). RTX also interferes with antigen presentation, modulates T cell proliferation, and affects natural killer cells and macrophage activities (141). Moreover, in recurrent FSGS, RTX appears to protect the renal filtration barrier through direct actions on podocytes (142).

Sustained depletion of CD27+ memory B cells and the return of naive B cells correlate with effective treatment (143). Delays in memory B cell reconstitution following RTX treatment may reduce relapse risks (35), underscoring that specific B cell subsets, rather than total numbers, drive disease pathology and treatment outcomes.

Advanced mechanistic insights reveal the complex interplay between traditional immunosuppressive approaches and emerging pathway-specific interventions. The gut-kidney axis modulation through microbiota restoration represents a novel therapeutic strategy where specific bacterial populations and their metabolites directly influence renal immune responses and disease progression (119). This mechanism demonstrates how probiotic therapy can be strategically integrated with conventional B cell-targeted approaches to achieve synergistic therapeutic effects.

Precision medicine approaches utilizing biomarker-guided therapy selection are becoming increasingly sophisticated. The identification of distinct immunological patterns and the application of network pharmacology approaches in traditional Chinese medicine provide unprecedented opportunities for developing combination therapies that target multiple disease mechanisms while minimizing treatment-related toxicities (136).

Future therapeutic directions incorporate multi-pathway targeting strategies that simultaneously address B cell dysfunction, complement activation, podocyte injury, and gut-kidney axis dysregulation. The integration of traditional Chinese medicine network pharmacology with modern immunological approaches offers unprecedented opportunities for developing combination therapies that target multiple disease mechanisms while minimizing treatment-related toxicities (119).

## 8 Fundamental differences in B-cell development and function between children and adults

### 8.1 Age-related characteristics of the distribution of B-cell subsets

The B-cell system in children is in a developmental stage and exhibits characteristics that are significantly different from those in adults (as shown in **Table 3**). Morbach et al. studied 261 healthy individuals of different ages and showed that the proportion of CD27^+^ memory B cells increased with age, gradually increasing from 4-8% in infancy to 30-40% in adults (144). Blanco et al. Analysis of the 609 healthy subjects (0-80) of peripheral blood B cell subsets, confirm CD24^hi^CD38^hi^ transitional CD19 + B cells (with functions of regulating) in children (especially under the age of 5) is significantly higher than that among adults, and gradually decline with age (145). Meanwhile, CD27^+^IgM^+^ marginal zone B cells and CD27^+^IgD^-^ class-switching memory B cells are low in children and only approach adult levels after puberty (145, 146).

**Table 3:** Comparison of normal reference values of B cell subsets between children and adults.

B lymphocyte subgroups	Children (≤18 years)	Adult (>18 years)
Lymphocytes	2128 (1849–2788)	2182 (1614–2462)
CD19^+^	304 (226–370)	165 (133–255)
CD27^-^IgD^+^	230 (171–293)	119 (92–199)
CD27^+^IgD^+^	24 (12–32)	19 (12–34)
CD27^+^IgD^-^	17 (10–29)	15 (10–31)
CD27^-^IgD^-^	10 (7–19)	5 (4-10)
CD24^++^CD38^++^	14 (10–24)	8 (5-13)
CD21^low^CD38^low^	6 (2–12)	10 (6-17)
CD24^-^CD38^++^	3 (1-4)	2 (1-3)

Data source: Morbach H et al. values for B cell subpopulations from infancy to adulthood (144).

### 8.2 Age differences in B-cell function

Differences in B cell function between children and adults are mainly shown in the following aspects:

1. Differences in antigen reactivity: Simon et al. reviewed that the response pattern of B cells in children to CD40L and TLR ligand stimulation is different from that in adults, which affects their activation threshold and effector function (147). The maturation process of affinity of B cells in children for T cell-dependent antigens is inefficient, reflecting that the function of the germinal center is not fully mature (147).
2. Cytokine production profile: Children’s B cells produce more IL-10 and less TNF-α, showing stronger regulatory potential (148). Levy et al. showed that neonatal and infant B cells respond to TLR agonists differently from adults, producing different patterns of cytokines (149).

### 8.3 Age-related characteristics of B-cell subsets in nephrotic syndrome

#### 8.3.1 Minimal change disease

B cell subsets of the literature character differences include: B cell number changes: Colucci et al. studied 54 children with MCD, found that disease activity dramatic decline in the number of peripheral blood CD19 ^+^ B cells is absolutely an average reduction of 50-60% (150). In contrast, the decrease of B cell number in adult MCD patients is relatively small (151). Plasma cell increased: the same study shows that children with MCD disease activity CD19 ^+^ CD24 ^-^ CD38hi pulp mother cell percentage increase (5.2 +/- 1.8% of healthy controls 1.8 +/- 0.6%), and urine protein levels were positively correlated (150).

#### 8.3.2 Membranous nephropathy

Membranous nephropathy membranous nephropathy in children is relatively rare, but more common in adults. According to the study of Beck et al., anti-PLA2R antibodies can be detected in the serum of about 70-80% of adult patients with primary MN, while this proportion is significantly reduced (about 15-20%) in children with MN (141). This reflects the differences in the etiology of MN in different age groups.

### 8.4 Effect of age-specific B-cell changes on treatment response

#### 8.4.1 B cells and prognostic markers of nephrotic syndrome in children

Current investigations into INS are exploring the unique B lymphocyte phenotypes associated with disease mechanisms and characteristics. Research suggests that CD24^high^CD38^high^ transitional B cells may serve as distinguishing markers between SSNS and steroid-resistant nephrotic syndrome (SRNS) in pediatric patients, with lymphocyte frequencies exceeding 2.05% indicative of SSNS (6). Additionally, researchers have identified that decreases in the transitional-to-memory B cell proportion could signal approaching relapses in SSNS patients (152). Clinical observations reveal elevated memory B lymphocyte populations in children with SSNS during initial diagnosis and relapse episodes when compared to both healthy controls and patients with steroid-refractory disease (6, 150). Following rituximab therapy, the timing of memory B cell repopulation—particularly switching memory B cells—correlates significantly with subsequent disease recurrence (35). Similarly, the interval required for CD20+ lymphocyte reconstitution post-rituximab administration shows association with remission longevity in steroid-dependent INS pediatric cases (153). More comprehensive characterization of memory B cell immunophenotypes may therefore facilitate the development of prognostic indicators for SSNS recurrence in patients undergoing B cell depletion treatments.

#### 8.4.2 Age-related response differences in B-cell targeted therapy

Rituxan in nephrotic syndrome shows effects in the treatment of children and adults, but the age-related differences: different treatment effects: Ravani et al. ‘s research show that the steroid-dependent children nephrotic syndrome patients treated with rituximab remission rate is significantly higher than adults (74% vs 56%) (154). Iijima et al. ‘s randomized controlled trial confirmed the efficacy of rituximab in children with frequent relapse/steroid-dependent nephrotic syndrome (155). Pharmacokinetic differences: children with rituximab clearance is faster than adults and need more frequent monitoring and possible dose adjustments (104). B cell reconstruction mode: The study found that children with rituximab B cells rebuild after the treatment process, unlike adults, initial B cells recover faster, slower recovery from memory B cells (156).

#### 8.4.3 Mechanistic explanations for these age-related treatment differences

##### 8.4.3.1 Immune system maturity differences

Enhanced B-cell plasticity in children: Pediatric B-cell compartments exhibit greater plasticity and adaptability compared to adults (147, 157). Children’s developing immune systems demonstrate higher responsiveness to immunomodulatory interventions, with B-cell reconstitution after rituximab depletion potentially favoring the generation of protective regulatory B-cells (158).

Superior regenerative capacity: Pediatric patients possess more active bone marrow hematopoiesis with abundant B-cell precursors, enabling faster and higher-quality B-cell reconstitution following rituximab-induced depletion (32, 159). This enhanced regenerative capacity may contribute to more durable therapeutic responses.

Age-related regulatory B-cell function: Adult regulatory B-cells may exhibit diminished immunosuppressive function due to immunosenescence, whereas pediatric Bregs maintain optimal regulatory capacity, facilitating better therapeutic rebalancing of immune homeostasis (147, 160).

##### 8.4.3.2 Disease pathological differences

1. Distinct pathological distributions: Pediatric nephrotic syndrome predominantly consists of idiopathic SSNS, with >90% being MCD (20, 161). In contrast, adult nephrotic syndrome presents with heterogeneous pathologies including MN, FSGS, and secondary forms (162, 163).
2. Pathogenetic mechanisms: MCD in children primarily involves T-cell-mediated podocyte dysfunction with less complex B-cell-mediated pathways (20, 164). Adult MN involves specific autoantibody production (anti-PLA2R, anti-THSD7A), while FSGS encompasses more complex pathogenetic mechanisms including genetic factors and structural abnormalities (162, 165).
3. Secondary disease burden: Adult patients frequently present with secondary nephrotic syndrome associated with comorbidities (diabetes, infections, malignancies), creating additional therapeutic challenges that may diminish rituximab efficacy (163, 166).

##### 8.4.3.3 Pharmacokinetic/pharmacodynamic considerations

1. Enhanced target sensitivity: Pediatric B-cells may exhibit more uniform and higher-density CD20 expression, potentially increasing sensitivity to rituximab-mediated complement-dependent cytotoxicity (CDC) and antibody-dependent cell-mediated cytotoxicity (ADCC) (167, 168).
2. Optimal drug exposure: Body surface area-based dosing in children may achieve more favorable rituximab concentrations compared to adults, where under-dosing concerns have been raised (169, 170). Population pharmacokinetic studies suggest that pediatric patients achieve adequate drug exposure with standard dosing regimens (171).
3. Reduced drug interactions: Pediatric patients typically have fewer concomitant medications, particularly fewer immunosuppressive agents, reducing potential drug interactions that might compromise rituximab efficacy (69, 172).
4. Enhanced tissue responsiveness: Pediatric kidneys may demonstrate superior responsiveness to B-cell depletion benefits, with enhanced podocyte repair capacity and reduced chronic structural damage compared to adult kidneys (20, 173).
5. Favorable clearance characteristics: While children may have faster rituximab clearance, this may actually prevent excessive immunosuppression and maintain appropriate immune balance, contributing to better therapeutic outcomes (171, 174).

These mechanistic insights explain why rituximab demonstrates superior efficacy in pediatric nephrotic syndrome and support the development of age-specific treatment protocols. The 18% higher response rate in children (74% vs 56%) reflects fundamental biological differences rather than mere statistical variation, providing a strong rationale for optimized pediatric dosing strategies and treatment approaches.

## 9 B cell subsets in disease monitoring of nephrotic syndrome

### 9.1 Pathological classification and diagnosis

B cell-related immunopathology offers crucial insights into the classification and differential diagnosis of NS. Advances in B cell subset analysis have enhanced our understanding of pathological subtypes and their immunological characteristics.

Immunofluorescence and immunohistochemistry reveal distinct B cell-related immune complex patterns across NS subtypes. In MN, granular IgG and C3 deposits along glomerular basement membranes indicate immunoglobulin-mediated autoimmunity (175), while unique IgG4 subclass compositions help differentiate primary from secondary forms (176). FSGS may present with mesangial IgM and C3 deposits (177), although their pathogenic significance remains debated. MPGN typically shows strong immunoglobulin and complement deposition, with monoclonal immunoglobulins potentially indicating monoclonal gammopathy of renal significance.

Flow cytometric analysis of peripheral blood B cell subsets provides additional diagnostic value. In primary MN, significantly elevated CD19+CD27+IgD- memory B cells correlate with disease-specific anti-PLA2R antibodies (178). FSGS patients exhibit increased CD19+CD27+IgD-IgG+ class-switched memory B cells, correlating with disease severity and renal function deterioration (22). Such B cell subset profiles may complement traditional histopathology in distinguishing FSGS subtypes and identifying patients likely to benefit from immunosuppressive therapy (179).

Integrating clinical parameters, traditional histopathology, and B cell phenotyping creates a comprehensive diagnostic framework, especially in challenging cases such as atypical presentations and differentiating minimal change disease from FSGS (35, 180). B cell markers may also help identify patients with immune-mediated components amenable to targeted therapy, even when histology does not support this (as shown in **Table 4**) (23).

**Table 4:** B lymphocyte markers in nephrotic syndrome.

Marker	CellType/Subset	Function	Relevance to Disease State
CD19	All B cells	Essential for B cell activation and signal transduction	Increased expression observed in nephrotic syndrome, indicating B cell activation (6, 35).
CD20	Mature B cells	Regulates B cell proliferation and differentiation	Elevated in active nephrotic syndrome, associated with ongoing immune responses (27, 32).
CD21	Follicular B cells	Acts as a receptor for complement, enhancing antigen recognition	Reduced expression noted in minimal change disease (MCD) and focal segmental glomerulosclerosis (FSGS) (33, 34).
CD24^hi CD38^hi	Transitional B cells	Involved in B cell development and activation	Increased in MCD and active lupus nephritis (6).
CD19^+^ CD24⁻ CD38^hi	Activated B cells	Indicative of plasmablast differentiation and activation	Strongly associated with MCD (150).
CD19^+^ CD27^hi CD38^hi	Plasma blasts	Highly differentiated B cells that secrete antibodies	Increased frequency in lupus nephritis, reflecting active immune responses (29).
CD19^+^ CD27^- IgD^-	Double negative B cell subset	Represents an atypical B cell population	Associated with autoimmune diseases, observed in lupus nephritis (104).
CD21^low CD38^low	Abnormal exhausted B cells	Indicative of B cell exhaustion	Elevation observed in lupus nephritis (129).
CD19^+^ CD24^hi CD38^hi	Regulatory B cells	Modulates immune responses and maintains homeostasis	Impaired regulatory B cell function reported in patients with lupus nephritis (29).
CD19^+^ CD27^+^ memory B cells	Memory B cells	Crucial for long-term immunity and sustained depletion of memory B cells	Increased levels suggest ongoing immune stimulation in diabetic nephropathy (240); elevation during initial onset and relapse in SSNS. Sustained depletion correlates with effective treatment; reappearance of naive B cells also noted (143). Delays in memory B cell reconstitution reduce relapse risk (35).
CD27+IgM+	Marginal zone B cells	Involved in rapid antibody responses	Increased in MCD (145).
CD27+IgD+IgM+	Naive and non-class-switched B cells	Indicates immature B cells	Presence in MN may reflect ongoing immune dysregulation (128).
CD27+IgD-IgG+	Class-switched memory B cells	Reflects maturation and class-switching	Increased levels associated with disease activity in FSGS (22); particularly elevated during initial onset and relapse in SSNS.
CD27+IgD-	Class-switching memory B cells	Indicates class-switching and memory formation	Studied in relation to MCD (21); significantly elevated CD19+CD27+IgD- memory B cells correlate with disease-specific anti-PLA2R antibodies in primary MN (178).
CXCR5	Follicular B cells	Regulates B cell migration to germinal centers	Changes in CXCR5 expression reported in diabetic nephropathy (106).
IgD	Naive B cells	Marker for B cell maturation	Altered IgD expression may reflect B cell development status in various nephropathies (145).
BAFF-R	B cells	Receptor for B cell activating factor (BAFF)	Dysregulation of BAFF-R noted in nephrotic syndrome, affecting B cell survival, observed in FSGS and diabetic nephropathy (78, 241).
CD19+ CD27^- IgD+	Naive B cells	Involved in the initial response to antigens	In acute MN, the proportion of naive B cells in peripheral blood increases, while switched and unswitched memory B cells decrease, patterns that closely correlate with proteinuria severity (93, 242).
Plasmablasts	Plasmablasts	Highly differentiated B cells that secrete large amounts of antibodies	Levels closely correlate with lower albumin levels, higher proteinuria, and hypogammaglobulinemia in relapsing MCD (56). Reappearance often precedes clinical relapse and elevation of anti-PLA2R antibodies in MN patients (32).
CD19-CD38++ Plasma Cells	Circulating plasma cells	Indicative of highly differentiated plasma cells	Detection helps identify early disease recurrence in MN patients, even when total B cell counts remain low (188).

### 9.2 B cell subsets in monitoring disease activity and prognosis

Flow cytometry enables precise identification of B cell subsets in various functional states, including resting naive B cells, regulatory B cells, and memory B cells (181). Dynamic changes in these subsets provide valuable insights into B cell functional status, disease activity, and prognostic trajectories in nephrotic syndrome patients (182).

The distribution and phenotype of B cell subsets correlate strongly with NS disease states. In acute MN, the proportion of naive B cells in peripheral blood increases, while switched and unswitched memory B cells decrease (93), closely correlating with proteinuria severity (128). Elevated B lymphocyte infiltration in renal tissue indicates worsening acute injury (183), as demonstrated by Du et al., who found positive correlations between renal B cell infiltration and disease severity (184).

In SSNS, B lymphocyte subsets undergo distinctive changes across disease phases, with total B cell counts and memory B cells (especially class-switched memory B cells) increasing during active disease. Research indicates that plasmablast levels in relapsing MCD patients correlate with low albumin levels, high proteinuria, and hypogammaglobulinemia (56). These findings highlight the potential of B cell subset analysis as a non-invasive method to monitor disease activity.

Longitudinal changes in B cell subsets provide critical prognostic information as well. Pediatric NS patients with elevated memory B cells at onset, particularly class-switched memory B cells, have observable recovery patterns following RTX treatment that can predict relapse (185). Colucci et al. noted that the timing and composition of B cell reconstitution after RTX strongly predict clinical outcomes (35). Increased CD27+IgD-IgG+ class-switched memory B cells in primary FSGS correlate with estimated glomerular filtration rate (eGFR), 24-hour proteinuria, and serum IgG levels, serving as sensitive biomarkers for disease severity (22). Meanwhile, regulatory B cells may indicate immune quiescence or future relapses (69).

The predictive value of B cell monitoring extends to treatment response assessment. During remission, total B cell counts and subset distributions normalize, while imbalanced reconstitution (especially elevated memory B cell proportions) following depletion therapy may indicate increased relapse risk (22). This differential pattern can guide individualized follow-up and treatment protocols.

### 9.3 Monitoring treatment response and optimizing strategies

Monitoring B cell subsets is vital for therapeutic decision-making in B cell-targeted therapies. While B lymphocyte depletion after RTX is expected, it alone does not guarantee clinical response (5, 186). Studies suggest that recovery of specific B cell subsets rather than total numbers predicts treatment outcomes better (35, 55).

Memory B cells are particularly significant; their sustained depletion corresponds with reductions in proteinuria (8), while early repopulation may indicate relapse risks (187). In pediatric NS, class-switched memory B cell reconstitution typically occurs before clinical relapse (185). Comprehensive immunophenotyping shows that long-term responders maintain lower levels of memory and switched memory B cells (35).

Plasmablast monitoring offers additional insights, as their reappearance often precedes clinical relapse (11) and anti-PLA2R antibody elevation in MN patients (188). Detecting circulating CD19-CD38++ plasma cells can help identify early disease recurrence, even when total B cell counts are low.

These findings have led to tailored monitoring protocols, where regular assessment of B cell subsets informs treatment strategies and optimal retreatment timing. Integrating B cell subset analysis with serum autoantibody monitoring (e.g., anti-PLA2R in MN) provides a comprehensive evaluation of immunological activity (23, 68).

Moreover, serial biopsies or novel non-invasive techniques to monitor tissue-resident B cells may further enhance treatment precision (183). Emerging biomarkers like BAFF levels and B cell cytokine signatures can identify patients at risk for inadequate response or early relapse (78, 189).

Despite their effectiveness, current B cell-targeted therapies have limitations, with a significant number of patients failing to achieve satisfactory proteinuria reduction due to individual variations, dosing differences, underlying disease heterogeneity, prior immunosuppression, and pharmacokinetic factors (64, 187, 190, 191). Current monitoring practices remain suboptimal, as routine assessments of CD19+ and CD20+ B cells show weak correlations with treatment response (132). Certain B cell subsets, particularly memory B cells, may evade RTX-mediated depletion or repopulate earlier, promoting relapse (192).

To address these issues, newer anti-CD20 agents, including ocrelizumab, ofatumumab, and obinutuzumab, are being explored, along with emerging therapeutic targets beyond CD20, such as Bruton’s tyrosine kinase inhibitors and proteasome inhibitors, which offer potential for precise interventions in B cell-mediated pathologies (193). Dynamic monitoring of treatment resistance patterns and assessing post-treatment B cell recovery characteristics will be essential for tailoring treatment strategies to each patient’s unique clinical profile.

## 10 Methodological heterogeneity challenges in B cell research for nephrotic syndrome

Significant methodological heterogeneity exists across B cell-related studies in NS, directly impacting the reliability, comparability, and clinical application of research findings. The following analysis examines key heterogeneity factors and their impact on evidence interpretation.

### 10.1 Patient population and phenotypic analysis differences

Existing studies show marked differences in patient population characteristics. Some studies focus on pediatric idiopathic NS patients, while others concentrate on adult patients or specific pathological types (such as MN, MCD, or FSGS). For example, research on pediatric idiopathic NS demonstrates specific patterns in B cell subset distribution (35, 185) (34, 56), while adult MN patients exhibit unique B cell characteristics associated with anti-PLA2R antibodies (134).

More importantly, B cell subset definitions and phenotyping strategies vary considerably. Earlier studies primarily monitored total CD19^+^ B cells (132, 133), while recent research employs more refined multi-marker combinations (56, 181). For instance, memory B cells are defined variously across studies as CD19^+^CD27^+^, CD27^+^IgD^+^/CD27^+^IgD^-^ (distinguishing non-switched and switched memory cells), or CD19^+^CD27^+^IgD^-^IgG^+^ (22, 185). These definitional differences directly influence measurement results and their correlation analyses with clinical parameters.

### 10.2 Technical and clinical assessment differences

Significant variations exist in flow cytometry technical parameters (such as instrument configuration, antibody clones, compensation strategies) and sample processing methods across studies (187, 194). More critically, sample collection timepoints relative to disease activity status (initial onset, relapse, or remission) and treatment intervention timing differ substantially (8, 56). For example, B cell reconstitution monitoring frequency after RTX treatment ranges from monthly to quarterly (35, 132), potentially missing critical features of subset recovery.

Additionally, clinical outcome assessment criteria lack consistency. Different studies employ varying methods of proteinuria assessment (24-hour urine protein vs. urine protein/creatinine ratio), remission definition thresholds, and follow-up durations (ranging from months to years) (116, 133), directly affecting result interpretation and inter-study comparisons.

### 10.3 Impact on research conclusions and recommendations

These methodological heterogeneities have led to several key contradictions in the literature, such as inconsistent reports on the correlation between B cell subsets and disease activity (181, 195), divergent observations regarding B cell recovery patterns (35, 143), and controversies surrounding treatment response predictors (177).

1. To enhance the reliability and comparability of future research, we recommend: Standardization of B cell subset definitions: Adopt unified phenotypic marker combinations that include at least CD19, CD27, CD38, and IgD to distinguish naïve, memory, non-switched, switched memory, and plasmablast subsets (181).
2. Standardized sample collection strategies: Implement normalized sampling at key timepoints throughout disease cycles and treatment processes, particularly for B cell-targeted therapies such as RTX (191).
3. Technical platform standardization: Establish unified flow cytometry protocols for multicenter studies to ensure data comparability (69).
4. Enhanced data sharing and integrated analysis: Promote original data sharing to support unified re-analysis of results from different studies (195).

Through methodological standardization, future research will provide more reliable and comparable evidence for the application of B cell analysis in NS diagnosis, prognosis assessment, and treatment monitoring.

## 11 Controversies of causality and correlation and limitations of the T-cell-centric paradigm

### 11.1 Controversies of causality and correlation

In the study of B cell subset changes, understanding the distinction between causality and correlation is crucial. Current literature indicates that there is a statistical correlation between B cell subset changes and disease progression in patients with kidney disease. For example, some studies have shown that the number of memory B cells and plasma cells significantly increases in NS, and these changes correlate positively with disease activity (56, 184, 196). Despite these observations providing important clinical clues, they do not establish that changes in B cell subsets are the direct cause of disease progression. This raises an urgent question for researchers: how should we interpret these observed correlations?

**Specific controversies include the following key questions:**

#### 11.1.1 Controversy over the causal direction of B cell subset changes

Are specific B cell subsets (such as elevated plasmablasts) the cause of podocyte injury, or are they a consequence of renal damage/inflammatory environment (or bidirectional)? The core of this controversy lies in determining whether B cell activation directly causes renal pathological changes or merely represents reactive proliferation in response to existing renal damage. Recent evidence suggests the possibility of bidirectional effects: on one hand, activated B cells may directly damage podocytes through the production of pathogenic antibodies and pro-inflammatory factors (197, 198). Campbell et al. demonstrated that autoimmune B cells may be the primary cause of nephrotic syndrome in some patients through the production of antibodies targeting podocyte antigens (197). On the other hand, the renal inflammatory environment may provide activation signals for B cells, forming a pathological positive feedback loop. Studies have shown that plasmablasts are significantly elevated in minimal change nephrotic syndrome with relapse, but whether this represents a cause or consequence remains debated (36, 38).

#### 11.1.2 Controversy over the mechanism of B cell depletion therapy

Does B cell depletion therapy (such as rituximab RTX) work primarily by eliminating pathogenic B cells (supporting causality), or through broader immunomodulation (indirect correlation)? The perspective supporting causality argues that RTX efficacy demonstrates the central role of B cells in disease pathogenesis (36, 199). Chan et al. noted that rituximab depletes all circulating B cell subsets, and its therapeutic efficacy highlights the key role of B cells in nephrotic syndrome pathogenesis (199). However, RTX may also affect T cell function, cytokine networks, and overall immune homeostasis beyond B cell depletion (200, 201). Munyentwali et al. suggested that rituximab therapy may be effective not only through CD20 lymphocyte depletion but also by modulating podocyte function (201). These broad immunomodulatory effects make it difficult to directly attribute therapeutic effects to B cell elimination alone.

#### 11.1.3 Controversy over the relationship between B cell reconstitution and disease relapse

Are observed B cell characteristics (such as memory B cell reconstitution) the cause of relapse or early signs of it? Although there is a strong correlation between the reappearance of memory B cells and disease relapse, this correlation does not necessarily indicate causation (35, 185). Colucci et al. demonstrated that relapses of nephrotic syndrome are associated with reconstitution of memory B cells, which reappear only after naïve B cells (35). However, Fribourg et al. reported temporal correlation between B cell reconstitution and relapse in some cases, while other patients remain in remission despite B cell recovery (185). Memory B cell reconstitution may merely be a marker of immune system recovery, while disease relapse may be triggered by other independent factors, with the two coincidentally appearing temporally (202).

The limitation of correlational studies is that even when a significant relationship between two variables is observed statistically, it does not necessarily mean that a change in one variable causes a change in the other. Chronic inflammation and autoimmune responses are important factors influencing B cell function, and these factors are often closely related to the progression of kidney disease. Research shows that changes in B cell subsets in patients with chronic kidney disease may reflect an adaptive response of the body to ongoing renal damage, rather than a direct mechanism leading to disease progression (203, 204).

These controversies highlight the necessity for further mechanistic studies, particularly the need to establish clear causal chains through longitudinal studies, animal models, and *in vitro* functional experiments to guide more precise therapeutic strategy development.

### 11.2 Limitations of the T-cell-centric paradigm

We plan to critically assess how these differences in B-cell behavior and function challenge conventional paradigms that currently dominate the understanding of nephrotic syndrome. This critical evaluation will focus on three key areas:

#### 11.2.1 Limitations of the T-cell-centric paradigm

The traditional T-cell-centric paradigm, established since Shalhoub’s 1974 hypothesis, faces several critical limitations that cannot adequately explain the full spectrum of nephrotic syndrome pathogenesis (70):

1. Incomplete disease coverage: The T-cell paradigm primarily explains minimal change disease (MCD) pathogenesis but fails to account for antibody-mediated diseases like membranous nephropathy, where specific autoantibodies (anti-PLA2R, anti-THSD7A) are the primary pathogenic drivers (205, 206).
2. Therapeutic response paradox: T-cell-targeted therapies show limited efficacy in many NS patients, while rituximab B-cell depletion demonstrates non-inferiority to cyclosporine with superior maintenance of proteinuria remission in membranous nephropathy and shows efficacy in pediatric steroid-dependent nephrotic syndrome, suggesting B-cells play more central roles than previously recognized (133, 154).
3. Autoantibody production mystery: The paradigm cannot explain the specific autoantibody production patterns observed across different NS subtypes, particularly the high specificity of anti-PLA2R antibodies for primary membranous nephropathy, and the recent discovery of anti-nephrin antibodies in some MCD patients (57, 205).

#### 11.2.2 Strong evidence supporting B-cell centrality

High-efficacy B-cell depletion therapy: The MENTOR trial demonstrated rituximab’s non-inferiority to cyclosporine in membranous nephropathy treatment, with superior maintenance of proteinuria remission (133). In pediatric steroid-dependent nephrotic syndrome, Ravani et al.’s multicenter randomized controlled trial confirmed rituximab’s effectiveness (154).

Autoantibody-driven pathogenesis: The discovery of specific autoantibodies has revolutionized our understanding:

1. Anti-PLA2R antibodies are present in approximately 70% of idiopathic membranous nephropathy patients (205).
2. Anti-THSD7A antibodies are found in approximately 10% of PLA2R-negative membranous nephropathy patients (206).
3. Anti-nephrin antibodies were recently discovered in approximately half of adult MCD patients and pediatric idiopathic nephrotic syndrome patients (57).
4. B-T cell interaction evidence: Emerging studies demonstrate complex bidirectional interactions where B-cells serve as antigen-presenting cells to activate podocyte-specific T-cells, while T-cells provide help for pathogenic antibody production (158, 207).

#### 11.2.3 Integrated new paradigm: the B-T cell immune axis

Rather than dismissing T-cell contributions, we advocate for a “B-T Cell Immune Axis” paradigm that positions:

1. B-cells as key effectors and regulators: B-cells function as both primary pathogenic drivers (through autoantibody production) and critical immune regulators (through cytokine secretion and T-cell modulation).
2. T-cells as essential collaborators: T-cells provide crucial help for B-cell activation and maintain chronic inflammatory responses, but their role is contextual rather than central.
3. Disease-specific mechanisms: Different NS subtypes exhibit distinct B-T cell interaction patterns:
4. MCD: Predominant T-cell-mediated with B-cell amplification, with some patients having anti-nephrin antibodies (57).
5. Membranous nephropathy: B-cell-driven autoantibody production with T-cell help (2, 3)
6. FSGS: Complex B-T cell crosstalk with tissue-specific factors

This paradigm shift is supported by the differential therapeutic efficacy of B-cell-targeted treatments, suggesting that B-cell-targeted precision medicine should be the cornerstone of future NS treatment strategies, with T-cell modulation as a complementary approach (69, 172).

## 12 Future perspectives and directions

### 12.1 Precise classification and functional mechanism analysis of B cell subsets

With the advancement of single-cell sequencing technology, future research should establish more precise B cell subset classification systems (63, 181). Through integrating multi-omics analysis strategies, we should deeply analyze the functional heterogeneity of key subsets such as transitional B cells, memory B cells, and regulatory B cells (36, 188), identify specific B cell clones driving disease progression, and elucidate the precise molecular mechanisms of B-T cell interactions (21).

### 12.2 Standardized B cell monitoring and precision diagnostic systems

Based on the specific alterations of B cell subsets in different pathological types revealed in this review, standardized B cell monitoring protocols should be established in the future (181). We should develop multidimensional disease activity assessment systems integrating B cell subset characteristics, functional states, and autoantibody profiles, particularly targeting key biomarkers for different types such as MCD, MN, and FSGS (22, 68, 185). Standardized flow cytometry analysis procedures (181) and novel point-of-care testing technologies should be established to provide precise guidance for clinical decision-making.

### 12.3 Next-generation B cell-targeted therapeutic strategies

Building upon existing rituximab therapy, future research should develop next-generation B cell-targeted drugs such as obinutuzumab, ofatumumab, and daratumumab (72, 193), exploring therapeutic strategies beyond CD20 targets. Individualized “precision dosing” protocols should be developed based on B cell subset monitoring results, guiding optimal treatment timing through memory B cell recovery patterns (35, 187). Meanwhile, combining emerging technologies such as chimeric antigen receptor T cell therapy provides additional therapeutic options for immune regulation.

### 12.4 Age-specific and systems biology research

Future studies should deeply explore age-related differences in B cell mechanisms between pediatric and adult nephrotic syndrome (144–146), establishing age-stratified treatment protocols (154, 156). Utilizing artificial intelligence and machine learning technologies, we should integrate multi-omics data to construct molecular networks of B cell-mediated diseases and establish disease stratification and treatment response prediction models. Multi-center prospective studies should validate the clinical application value of B cell biomarkers (195).

Through in-depth exploration of these research directions, we expect to significantly enhance our understanding of B cell mechanisms in nephrotic syndrome within the next 5–10 years, translating these findings into precision diagnostic and individualized therapeutic strategies to ultimately improve patient prognosis and quality of life.

### 12.5 Multi-omics integration and artificial intelligence applications

Utilizing artificial intelligence and machine learning technologies will facilitate the integration of multi-omics data, allowing for the construction of comprehensive molecular networks related to B cell-mediated diseases. These networks can aid in the development of disease stratification and treatment response prediction models by integrating key biomarkers:

1. B-cell subset profiling: Memory B-cell (CD27+) populations predict early relapse risk, while regulatory B-cell dysfunction indicates the need for adjunctive therapy (208).
2. Autoantibody-guided treatment: Anti-PLA2R, anti-THSD7A, and anti-nephrin antibodies guide treatment intensity and duration (57, 205).
3. Machine learning prediction: AI algorithms integrating clinical and immunological data significantly enhance treatment selection precision (209).

### 12.6 To address the knowledge gap regarding whether changes in B cell subsets are part of the etiology, future research should focus on the following key areas

#### 12.6.1 Longitudinal tracking studies

Design longitudinal studies to observe the relationship between changes in B cell subsets over time and the course of kidney disease, attempting to construct causal chains. For example, by regularly monitoring B cell subsets in patients with kidney disease along with clinical indicators (such as renal function and disease activity), a clearer identification of the temporal relationships between B cell changes and disease progression can be achieved (204). Advanced statistical methods, such as mediation analysis, regression discontinuity analysis, and Mendelian randomization, can also be employed to further explore the causal relationship between B cell subsets and disease activity in nephrotic syndrome.

#### 12.6.2 Interventional experiments

To establish a causal relationship between B cell subset changes and NS progression, we propose designing systematic interventional experiments through both animal models and clinical trials to validate our hypotheses. In animal experiments, we should select classic models that effectively recapitulate different pathological types of human NS, including adriamycin-induced nephropathy (mimicking FSGS-like lesions) (210), passive Heymann nephritis (mimicking membranous nephropathy) (211), and puromycin aminonucleoside model (mimicking minimal change disease) (212). The selection of these models covers the major pathological phenotypes of NS, providing a foundation for studying the role of B cells in different disease environments.

Based on these animal models, B cell subset functions can be specifically manipulated through multiple strategies. Genetic engineering approaches include using CD20-Cre mice to achieve conditional B cell depletion and utilizing AID knockout mice to block antibody class switching processes (213). Pharmacological interventions can achieve different degrees of B cell depletion through anti-CD20, anti-CD19, or anti-BAFF antibodies (69). Additionally, adoptive transfer experiments can directly assess the pathogenic potential of specific B cell subsets by transferring different B cell subpopulations from diseased animals to healthy recipients and observing whether corresponding renal lesions can be induced (214). The core observation endpoints of these experiments should encompass proteinuria levels, renal histological changes, renal function parameters, as well as mechanistic aspects including autoantibody production and inflammatory cytokine release (215).

At the clinical research level, existing B cell-targeted therapies provide valuable “natural experiment” opportunities. Rituximab, as a CD20 monoclonal antibody, can effectively deplete circulating B cells, and we can reveal its therapeutic mechanisms by tracking detailed dynamic changes in B cell subsets before and after treatment (121, 216). We recommend using high-throughput flow cytometry to analyze changes in numbers and functional states of naïve B cells, memory B cells, and plasma cells at key treatment timepoints (baseline, 1, 3, 6, 12, and 24 months). Belimumab, as a BAFF inhibitor, has a different mechanism of action from rituximab, primarily affecting the survival of BAFF-dependent B cells. By comparing the action patterns of these two drugs, we can better understand the specific functional roles of B cells in NS (217).

To obtain more precise research results, clinical trials should conduct detailed subgroup analyses, stratified by patient age (pediatric vs. adult), histological classification (minimal change disease, focal segmental glomerulosclerosis, membranous nephropathy), baseline B cell characteristics (high vs. low memory B cell proportions, autoantibody titers), and genetic backgrounds. This stratification strategy helps identify which patient populations are most likely to benefit from B cell-targeted therapy, thereby providing evidence for individualized treatment approaches.

#### 12.6.3 Multidisciplinary research

With the rapid development of high-throughput omics technologies, multi-omics integrative analysis has become an important approach for in-depth understanding of complex disease mechanisms. In studying the relationship between B cells and NS, we need to systematically integrate genomics, transcriptomics, proteomics, and metabolomics data to construct comprehensive B cell functional regulatory networks (218).

Genomics research should focus on genetic variants affecting B cell function. HLA system polymorphisms directly influence antigen presentation processes, thereby regulating B cell activation thresholds; complement system gene variants (such as C3, C4, CFH) may affect B cell activation and immune complex clearance; in African ancestry populations, APOL1 gene variants may modulate interactions between podocytes and B cells (219). By integrating genome-wide association study (GWAS) data with B cell-specific expression quantitative trait loci (eQTL) information, we can identify functionally significant genetic variants, providing a genetic basis for understanding inter-individual differences in B cell function.

Transcriptomic analysis, particularly single-cell RNA sequencing technology, provides unprecedented resolution for observing dynamic changes in B cells during disease progression (219, 220). By tracking the complete trajectory of B cell differentiation from naïve states to memory cells and plasma cells, we can identify key transcriptional regulatory programs, such as PRDM1, XBP1, and IRF4 controlling plasma cell differentiation, and BCL6 and BACH2 maintaining memory B cell characteristics. The dynamic change patterns of these molecular markers can help us understand the specific mechanisms of B cell action in NS.

Proteomic analysis focuses on the ultimate manifestations of B cell function. Through high-dimensional protein detection technologies such as mass cytometry, we can simultaneously detect expression levels of cell surface markers and secreted factors (221, 222). Key proteins of focus include cytokines regulating B cell function (IL-4, IL-6, BAFF, APRIL), autoantibodies against podocyte antigens, and complement proteins involved in immune complex formation and clearance. Changes in these protein levels directly reflect the functional states and pathogenic potential of B cells.

Metabolomic research reveals another important dimension of B cell functional regulation. B cells undergo significant metabolic reprogramming during activation, shifting from oxidative phosphorylation in the resting state to glycolytic metabolism, while amino acid and lipid metabolism are also adjusted accordingly to support antibody synthesis and cell proliferation (223, 224). By analyzing these metabolic change patterns, we can better understand how microenvironmental factors influence B cell function.

Integration of multi-omics data requires advanced computational biology methods. Pathway enrichment analysis (such as GSEA and Reactome databases) can identify common regulatory pathways across omics layers; weighted gene co-expression network analysis (WGCNA) can discover B cell-specific molecular modules; while machine learning methods such as multi-omics factor analysis (MOFA) and similarity network fusion (SNF) can integrate different omics data to identify comprehensive biomarker signatures associated with treatment response and disease progression (225). This systematic integrative analysis strategy will provide us with a panoramic understanding of B cell roles in NS.

#### 12.6.4 Influence of external factors

Explore how external factors (such as infections, inflammation, medications, etc.) influence changes in B cell subsets and lead to differences in clinical presentations among patients. For instance, certain infections may activate B cells, which in turn could affect disease progression; thus, a thorough analysis of the interactions between these factors is crucial for understanding the role of B cells (226).

## 13 Conclusions

This review reveals the central role of B lymphocyte subsets in nephrotic syndrome pathogenesis, with different pathological types of NS exhibiting distinct B cell subset alteration patterns that serve as important biomarkers for disease activity and prognosis. The remarkable efficacy of B cell-targeted therapies further confirms their pivotal position in the disease, while significant differences exist between primary and secondary NS as well as among different age groups in B cell mechanisms and therapeutic responses. With the development of emerging technologies and establishment of standardized platforms, in-depth analysis of B cell subset characteristics will provide new opportunities for precision diagnosis and individualized therapy of nephrotic syndrome, ultimately achieving precision medicine goals.
